# Structural deficits in key domains of Shank2 lead to alterations in postsynaptic nanoclusters and to a neurodevelopmental disorder in humans

**DOI:** 10.1038/s41380-022-01882-3

**Published:** 2022-11-30

**Authors:** Fatemeh Hassani Nia, Daniel Woike, Isabel Bento, Stephan Niebling, Debora Tibbe, Kristina Schulz, Daniela Hirnet, Matilda Skiba, Hans-Hinrich Hönck, Katharina Veith, Christian Günther, Tasja Scholz, Tatjana Bierhals, Joenna Driemeyer, Renee Bend, Antonio Virgilio Failla, Christian Lohr, Maria Garcia Alai, Hans-Jürgen Kreienkamp

**Affiliations:** 1 Institute for Human Genetics, University Medical Center Hamburg Eppendorf, Hamburg, Germany; 2 EMBL Hamburg, c/o DESY, Hamburg, Germany; 3 Centre for Structural Systems Biology, Hamburg, Germany; 4 Institute of Cell and Systems Biology of Animals, University of Hamburg, Hamburg, Germany; 5 Department of Pediatrics, University Medical Center Eppendorf, Hamburg, Germany; 6 Prevention Genetics, Marshfield, WI USA; 7 UKE microscopic imaging facility (umif), University Medical Center Eppendorf, Hamburg, Germany; 8 Present Address: III. Department of Medicine, University Medical Center Hamburg-Eppendorf, Hamburg, Germany

**Keywords:** Autism spectrum disorders, Cell biology

## Abstract

Postsynaptic scaffold proteins such as Shank, PSD-95, Homer and SAPAP/GKAP family members establish the postsynaptic density of glutamatergic synapses through a dense network of molecular interactions. Mutations in *SHANK* genes are associated with neurodevelopmental disorders including autism and intellectual disability. However, no *SHANK* missense mutations have been described which interfere with the key functions of Shank proteins believed to be central for synapse formation, such as GKAP binding via the PDZ domain, or Zn^2+^-dependent multimerization of the SAM domain. We identify two individuals with a neurodevelopmental disorder carrying de novo missense mutations in *SHANK2*. The p.G643R variant distorts the binding pocket for GKAP in the Shank2 PDZ domain and prevents interaction with Thr(−2) in the canonical PDZ ligand motif of GKAP. The p.L1800W variant severely delays the kinetics of Zn^2+^-dependent polymerization of the Shank2-SAM domain. Structural analysis shows that Trp1800 dislodges one histidine crucial for Zn^2+^ binding. The resulting conformational changes block the stacking of helical polymers of SAM domains into sheets through side-by-side contacts, which is a hallmark of Shank proteins, thereby disrupting the highly cooperative assembly process induced by Zn^2+^. Both variants reduce the postsynaptic targeting of Shank2 in primary cultured neurons and alter glutamatergic synaptic transmission. Super-resolution microscopy shows that both mutants interfere with the formation of postsynaptic nanoclusters. Our data indicate that both the PDZ- and the SAM-mediated interactions of Shank2 contribute to the compaction of postsynaptic protein complexes into nanoclusters, and that deficiencies in this process interfere with normal brain development in humans.

## Introduction

Shank/ProSAP proteins, encoded by *SHANK1*-*3* genes, are master scaffold proteins of the postsynaptic density (PSD) that link glutamate receptors to the actin cytoskeleton by integrating intermediate scaffolding proteins in dendritic spines. They share a set of domains including a Shank/ProSAP N-terminal (SPN) domain, a set of multiple ankyrin repeats (Ank), a Src homology 3 (SH3) domain, a PSD-95/discs large/ZO1 (PDZ) domain, a long Proline-rich region and a C-terminal sterile alpha motif (SAM) domain [1, 2]. The Shank PDZ domain constitutes the binding site for the PDZ ligand of SAPAP/GKAP proteins (termed GKAP from here on) that mediates an indirect interaction between Shank proteins and the NMDA receptors through binding to postsynaptic scaffold proteins of the PSD-95 family [3, 4]. Actin binding proteins cortactin, IRSp53 and Abi-1 [5–10] as well as Homer, an interaction partner of the metabotropic glutamate receptors (mGluRs) and IP3 receptors [11, 12], bind to the proline-rich region of Shank proteins. In the presence of Zn^2+^, the SAM-domain polymerizes into helical fibers, which may be stacked side-by-side through interhelical contacts. This enables the formation of large sheets where Shank proteins multimerize, allowing Shank proteins to serve as building blocks in deeper layers of the PSD [1, 13].

Shank proteins are concentrated in so-called nanoclusters, together with several direct and indirect interaction partners (Homer, GKAP proteins, PSD-95). It is assumed that nanoclusters serve to perfectly align the presynaptic release machinery with postsynaptic receptor-associated complexes [14, 15]. So far, it is unclear which principles govern their assembly.

Loss-of-function mutations [16–22] as well as rare missense variants [23–28] in all three *SHANK* genes are associated with neurodevelopmental disorders. Interestingly, the most widely studied functional domains of Shank proteins, such as the PDZ and SAM domains, and the cortactin and Homer binding sites are not functionally affected by missense mutations. Only in *SHANK2*, missense mutations have been reported which alter residues close to, or in the PDZ domain: S610Y; N690S in schizophrenia cases [23], and V717F in autism [20]. However, a functional effect on the PDZ-mediated interaction has not been analyzed. Consequently, the role of these core interaction motifs of Shank proteins in the pathogenesis of neurodevelopmental disorders is unclear.

Here, we report two patients with de novo missense mutations in *SHANK2*, p.G643R and p.L1800W, which affect two key functional domains of the Shank2 protein, the PDZ and the SAM domain, respectively. Both variants disrupt the molecular interactions of the respective domains. We provide structural explanations for these deficits through homology modeling and crystal structure analysis. Both variants interfere with the targeting of Shank to dendritic spines and alter synaptic transmission. Moreover, the L1800W mutation decreased the number of dendritic clusters positive for Shank2. STED microscopy revealed that both mutants affect the nanoscale organization of PSD by decreasing the number of Shank2 nanoclusters at synapses.

## Materials and methods

### Patients and genetic analysis

We report two patients affected by a neurodevelopmental disorder. Informed consent for genetic analysis was obtained from parents, and genetic analysis was performed as approved by the Institutional Review Board (approval number by the Ethics Committee of the Hamburg Chamber of Physicians: PV 3802). Genetic testing was performed by trio IDEA (Intellectual Disability, Epilepsy, and Autism) panel analysis, or trio whole-exome sequencing of the patients and parents. In both cases a heterozygous, de novo missense mutation was identified in patients. Clinical and molecular findings for patient 1 are summarized in the Supplementary Data, and in Table S1. The contact to the family of patient 2 was lost during follow-up; therefore, no protected health information (PHI) for this patient is included in this paper.

### Homology modeling

*Modeling* of the extended PDZ domain of Shank2 was performed using the Swissmodel server (swissmodel.expasy.org), using pdb entry 5IZU as a template.

### Expression constructs

A construct coding for mCherry-tagged human Shank2a was obtained from Dr. Simone Berkel (Heidelberg, Germany) [29]. The Shank2a cDNA was cloned into the pHAGE-GFP vector for transfecting neurons. A rat cDNA for CortBP1 (Shank2b), donated by Dr. J. Thomas Parsons (University of Virginia) was cloned into pEGFP-C2, leading to expression of N-terminally GFP-tagged Shank2b. cDNA coding for GKAP was obtained from Prof. Stefan Kindler (UKE, Hamburg). Mutations were introduced using the QuikChange II site-directed mutagenesis kit (Agilent). cDNA coding for the 50 C-terminal residues of GKAP was amplified by PCR with appropriate primers and subcloned into BamHI/EcoRI sites of pGEX-4T2, allowing for expression as a GST fusion. For expression of the Shank2-SAM domain, cDNA coding for residues T1780–R1849 was cloned into pET-SUMO (Thermo-Fisher), allowing for expression of a SUMO-SAM fusion with an N-terminal His_6_-tag. A cDNA-fragment coding for SH3 to PDZ domains of Shank2 (R520–D727) was cloned into pET-SUMO. shRNA constructs were generated in pSuper based on constructs used by Berkel et al. [29] carrying target sequences GGATAAACCGGAAGAGAT (shShank2#1) and GGAATTGAGCAAAGAGATT (shShank2#2). A construct coding for rat Homer1b with an N-terminal GFP-tag in pEGFP-C1 was described [30].

### Cell culture and transient transfection

293T HEK (human embryonic kidney; ATCC) cells were maintained in Dulbecco’s modified Eagle’s medium containing 10% fetal bovine serum and 1% penicillin/streptomycin. Cells were transiently transfected with Turbofect Transfection Reagent (Thermo Scientific) according to the manufacturer’s instructions. Cells were regularly monitored for absence of mycoplasma.

### Immunoprecipitation and pulldown experiments from transfected cells

Transfected cells were lysed in immunoprecipitation (IP) buffer (50 mM Tris-HCl, pH 8, 120 mM NaCl, 0.5% NP40, 1 mM EDTA), followed by centrifugation at 20,000 × *g* for 15 min at 4 °C. Immunoprecipitation of GFP-tagged proteins from supernatants was performed using 20 μl of GFP-trap beads (Chromotek, Munich, Germany). For pulldown assays using the extended PDZ ligand of GKAP, 50 μl of GST-GKAP fusion protein coupled to glutathione sepharose beads were used. Cell lysates were incubated with the respective beads for 2 h at 4 °C on a rotator. After washing, precipitates and input samples were processed for Western blotting.

### SDS PAGE and Western blot

Proteins were separated on SDS-PAGE under denaturing conditions and transferred to nitrocellulose membrane using a MINI PROTEAN II^TM^ system (Bio-Rad). Membranes were blocked with 5% milk powder/TBS-T and incubated with the primary antibodies overnight at 4 °C, followed by washing in TBS-T and then HRP-linked secondary antibodies at room temperature for 1 h. Membranes were scanned using a ChemiDoc^TM^ MP Imaging System (Bio-Rad) and images were processed and further analysed using Image Lab Software (Bio-Rad).

### Protein purification

GST fusion proteins were expressed in BL21(DE3) cells (Thermo-Fisher) and purified from bacterial lysates in STE buffer, using glutathione sepharose beads. Proteins were left on the beads and after several washing steps used for pulldown assays. His_6_-SUMO-tagged fusion proteins were expressed in BL21(DE3) cells and purified from bacterial lysates prepared in native lysis buffer (50 mM NaH_2_PO_4_, 500 mM NaCl, pH 8.0) using Ni–NTA agarose (Qiagen, Hilden, Germany). Proteins were eluted from beads with 250 mM imidazole in lysis buffer and immediately applied to G-25 columns (GE Healthcare), followed by elution in 0.5 M NaCl, 20 mM Tris/HCl pH 7.5 (for Zn^2+^ aggregation assays) or 150 mM NaCl, 50 mM Tris-HCl; pH 8.0 (for cleavage with SUMO protease). For protease cleavage, 1 mM DTT and 50 μl SUMO protease (Thermo-Fisher) were added to a total of 10 ml solution with a protein concentration of about 1 mg/ml. After digestion at 4 °C overnight, the His_6_-tagged SUMO part of the fusion protein, as well as His_6_-tagged SUMO protease, were removed by a second incubation with Ni-NTA Agarose. Efficiency of all protein purifications was verified by SDS-PAGE, followed by Coomassie staining. Protein concentrations were determined by Bradford assay, using BSA as a standard.

### Isothermal titration calorimetry (ITC)

Synthetic peptides corresponding to the complete PDZ ligands of GKAP (sequence: NH2-ADSIEIYIPEAQTRL-COOH) and CIRL1 (NH2-PGPDGDGQMQLVTSL-COOH) were obtained from Genscript (Leiden, The Netherlands). Affinities between WT and mutant Shank2 protein fragments (His_6_-SUMO-tagged SH3 to PDZ domains) and the peptides were measured by ITC using a MicroCal PEAQ-ITC instrument (Malvern Panalytical). All titrations were performed at 25 °C with protein concentrations between 20 and 30 µM (WT: 20 µM, G643R: 22 µM, N690S: 30 µM, S610Y: 25 µM, V717F: 27 µM) in the sample cell. The peptide concentration in the syringe was 200 µM (1 mM for the G643R variant). All solutions contained the same buffer (50 mM Tris-HCl, pH 8.0, 100 mM NaCl, 1 mM EDTA). For each titration, one initial injection of 0.4 µl (0.8 s injection time) followed by 12 injections with 3 µl each (6 s injection duration) with a spacing of 300 s between injections and a stirring speed of 500 rpm was done. All thermograms were baseline corrected and integrated using NITPIC [31]. SEDPHAT [32] was used for fitting a 1:1 binding model and GUSSI [33] for generating the final plots.

### Zn^2+^-dependent aggregation assays

Purified SAM domains were treated with different concentrations of Zn^2+^. 20 µl drops were placed on microscopy slides and the formation of aggregates was observed under the light microscope for 5 to 15 min. Clusters were photographed using a standard digital camera. For turbidity assays, isolated SAM domains were mixed in wells of a 96-well plate with varying concentrations of Zn^2+^ in a total volume of 200 µl. Absorbance at 350 nm was determined at different time points using Epoch™ Multi-Volume Spectrophotometer System [34].

### Protein crystallization and crystal structure determination

Crystals of the Shank2-SAM WT and mutant (L1800W) were obtained using the vapor diffusion method and sitting drop technique. SAM-WT crystals grew after 2 weeks from a crystallization condition containing 0.1 M Bis-Tris pH 5.5 and 0.3 M of Mg-formate and a protein concentration of 7.5 mg/ml. The mutant crystals appeared in 0.1 M Bis-Tris pH 5.5 and 26% polyethylene glycol monomethyl ether 2000 (PEG 2000MME), with a protein concentration of 11 mg/ml, after 48 h and took 14 days to grow to suitable size. X-ray data collected at the EMBL PETRA III beamlines (P14, P13) in Hamburg showed that SAM-WT and SAM-L1800W crystals diffracted to 2.1 Å; and 1.95 Å, respectively.

X-ray data sets were processed and scaled using XDS [35] and AIMLESS [36], respectively. Both crystal structures, SAM-WT and SAM-L1800W, were solved by the molecular replacement method, with program MOLREP [37], using as a search model the homologous structure of Shank3-SAM domain (PDBID 2f44) [13] for SAM-WT, and the refined structure of SAM-WT for the L1800W mutant structure. Structure refinement proceeded using REFMAC [38] or Phenix [39] programs and model building was done using COOT [40]. CCP4i2 [41] and ccp4cloud (https://cloud.ccp4.ac.uk/) were used during structure solution and refinement. Both structures were checked periodically using the PDB_redo server (https://pdb-redo.eu/) [42] and validated with MOLPROBITY [43]. The final structures were deposited in the Protein data bank (PDB, https://www.ebi.ac.uk/pdbe). Data collection and Refinement statistics are listed in Table S2. Figures were drawn with PyMOL (https://pymol.org/2/).

### Dynamic light scattering (DLS)

Measurements were performed using DynaPro Nanostar (Wyatt Technology Corporation). Data were processed using Dynamics v.7 software. 50 μM protein and 50 µM Zn^2+^ were mixed and incubated for 1 h at room temperature. Samples were filtered through 0.22 μm centrifugal filters (Millipore). The acquisition time was 3 s with a total of 30 acquisitions.

### Animals

Primary cultures of hippocampal neurons were prepared from *Rattus norvegicus* embryonic day-18 rats of both sexes (Envigo), as described [44]. All animal experiments were approved by, and conducted in accordance with, the guidelines of the Animal Welfare Committee of the University Medical Center Hamburg-Eppendorf (Hamburg, Germany) under permission number Org766.

### Neuron culture and transfection

After dissection of hippocampal tissue, neurons were extracted by enzymatic digestion and mechanical dissociation. Dissociated neurons were plated on glass coverslips and maintained in Neurobasal medium supplemented with 2% B27, 1% Glutamax and 1% Penicillin/Streptomycin. Transfection of neurons was performed at DIV7 using the calcium phosphate method. For Zn^2+^ treatment, 10 µM Zn^2+^ was added to transfected neurons (DIV8).

### Immunocytochemistry

Neurons (DIV10; DIV11 after Zn^2+^ treatment; DIV14 for Shank3 staining) were fixed with 4% paraformaldehyde in PBS for 15 min and permeabilised with 0.1% Triton X-100 in PBS for 5 min. After 1 h blocking with 10% horse serum in PBS, cells were incubated with corresponding antibodies overnight followed by washing and then 1 h of incubation with Alexa Fluor coupled secondary antibodies. The coverslips were mounted onto glass microscopic slides using ProLong^TM^ Diamond Antifade mounting medium.

### Microscopy

Confocal images were acquired with a Leica Sp5 confocal microscope using a 63x objective. Super-resolution imaging was performed using a STED microscope from Abberior. The STED images were acquired with the pixel size of 20 nm and the scanning area of ~15 µm. Quantitative analysis for images was performed using ImageJ. Primary dendrites were counted at a ring within 10 µm distance from the soma. The counting of clusters along dendritic branches was performed using the Multi-Point tool of ImageJ. The distance between the head of a dendritic spine and the dendritic shaft was measured using ImageJ’s straight line tool.

### Antibodies

The following primary antibodies were used: mouse anti-GFP (Covance MMS-118P-500, RRID: AB_291290; WB: 1:3000); rat anti-RFP (Chromotek 5F8, WB: 1:1000); rabbit anti-GFP (Abcam AB6556; ICC: 1:500); chicken anti-MAP2 (Antibodies-Online ABIN361345, ICC: 1:1000); mouse anti PSD-95 (Thermo-Fisher MA1-046; ICC: 1:500); guinea pig anti-Shank3 (Synaptic Systems 162 304; ICC 1:1000); rabbit anti-Shank2 (Sigma-Aldrich HPA008174; ICC 1:250); rabbit anti-VGlut (Synaptic Systems; ICC: 1:2000). HRP-labeled goat secondary antibodies were from Jackson ImmunoResearch and used for WB at 1:2500 dilution. For ICC, Alexa 405 goat anti-chicken IgG (abcam; ab175675 at 1:1000 dilution), Cy3 goat anti-rb IgG (Dianova; at 1:1000 dilution). Abberior STAR RED goat anti-mouse IgG and Abberior STAR RED goat anti-rabbit IgG (Abberior) were used at 1:1000 dilution.

### Recording of miniature excitatory postsynaptic currents

The synaptic activity of transfected primary hippocampal neurons was recorded at DIV 9-10, 2–3 days after transfection. Throughout the experiments, cells were superfused with ACSF (artificial cerebro spinal fluid) of following composition (in mM): NaCl, 120; NaHCO_3_, 26; NaH_2_PO_4_, 1; KCl, 2.5; Glucose, 2.8; CaCl_2_, 2; MgCl_2_, 1. The whole cell configuration was employed using patch pipettes with a resistance of 3–6 MΩ. Recordings were made using MultiClamp 700B and 700A amplifiers and Clampex 10.7 software (both Molecular Devices, LLC. San Jose, CA, USA). Recordings were digitized at 10 kHz and filtered (Bessel filter, 2 kHz). The pipette solution contained (in mM): CsCl, 120; Hepes, 10; EGTA, 0.2; MgCl_2_, 2; CaCl_2_, 0.075; Na-ATP, 2; Na-GTP, 0.5; 4-AP, 5; TEA-Cl, 20. To block voltage gated sodium channels and GABA_A_ receptors, 0.5 µM TTX and 5 µM gabazine were added to the ACSF. Cells were held at −65 mV in voltage clamp recording. Series resistance (R_series_) was monitored during the recording and cells exceeding 20 MΩ and/or a change of 20% in R_series_ were excluded from the analysis. MiniAnalysis software (Synaptosoft) was used to analyze frequency and amplitude of spontaneous synaptic events (mEPSC) which were collected over a 2 min period. The automated threshold function based on noise detection included in the software was applied before detection of events.

### Evaluation of data

Sample size was chosen in each case based on previous experience with the relevant technique [28, 44–47]. No samples were excluded. No randomization was used. For confocal analysis, samples were blinded for quantitative evaluation of measurements. Normal distribution of data, and statistical significance was determined using Prism8 software (GraphPad, San Diego, CA). Data were analysed by Student’s t-test or one-way ANOVA with post hoc Dunnett’s test or two-way ANOVA with post hoc Sidac’s and Dunnett’s test for normal distributed data. Otherwise, the Kruskal–Wallis test was used. For mEPSC analysis, the cumulative probability of inter-event intervals was plotted and tested for significant differences (Kolmogorov–Smirnov test) using OriginPRO software (version 2021b, Origin Lab Corp., Northampton, MA, USA). All data are presented as mean ± SD.

## Results

### Missense mutations in *SHANK2* cause a severe neurodevelopmental disorder

**Fig. 1:**
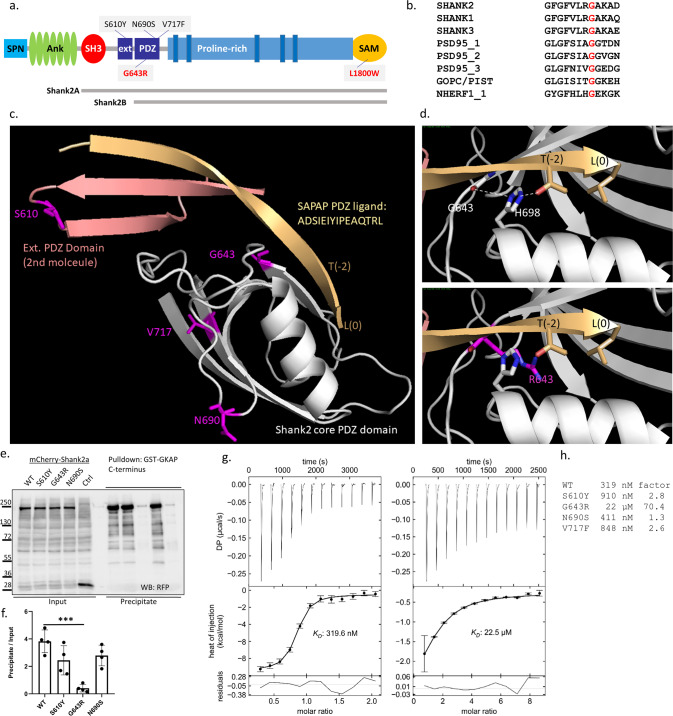
Mutations affecting key domains of *SHANK2*. **a** Domain structure of a generic Shank protein, ranging from SPN to SAM domains. The positions of variants in Shank2 PDZ and SAM domains are indicated (black for previously reported variants, red for variants reported in this study). Transcript variants Shank2a and Shank2b were used in this study and are indicated**. b** Alignment of the sequence around G643 in Shank2 with that of other type I PDZ domains. **c** Model of the extended PDZ domain of Shank2 in complex with the C-terminal PDZ ligand of GKAP/SAPAP1. Modeling is based on the structure of the Shank3/GKAP complex (5IZU) [50]. The extended binding surface is formed by two Shank2 molecules, one of which (light gray) provides the core PDZ domain, whereas the second molecule provides the extended sequence (salmon). The C-terminal GKAP sequence forms an extended β-sheet first with the extension part provided by the second molecule, and then with a β-strand of the core PDZ domain. The C-terminal leucine L(0) and the threonine at position (−2) of the PDZ ligand are indicated. S610, G643, N690 and V717 are indicated in magenta. **d** Upper panel. Magnification of the binding site in the core PDZ domain. G643 is located at the C-terminal end of the β-strand which is in contact with the PDZ ligand. Its carbonyl group forms an H-bond (white dashed line) to N1 of the conserved histidine H698; N2 of H698 makes another H-bond to the side chain oxygen of T(−2). Lower panel. Model of the G643R mutant protein. The introduction of the bulky arginine at position 643 (magenta) shortens the β-strand, and slightly displaces it upwards. In addition, R643 clashes with the side chain of H698 and pushes it to the left. This displacement is predicted to disrupt the interaction of H698 with T(−2) of the PDZ ligand. **e** mCherry-tagged Shank2a variants were expressed in 293T cells. Cell lysates were subjected to pulldown with a GST fusion protein of GKAP C-terminus. Input and precipitate samples were analyzed by Western blotting using anti-RFP antibodies. **f** Quantification of precipitation; ***, significantly different from WT, *p* < 0.001; data from four independent experiments; ANOVA, followed by Dunnett’s multiple comparison test. **g** Isothermal titration calorimetry of purified His_6_–SUMO tagged fusion protein containing SH3 to PDZ domains of Shank2 WT (left) and G643R mutant (right) vs. the synthetic peptide ADSIEIYIPEAQTRL which corresponds to the C-terminus of GKAP/SAPAP proteins. **h** Comparison of binding affinities for all PDZ domain mutants. The factor on the right is calculated as the ratio of Kd values for mutant divided by WT. ITC curves for the mutants S610Y, N690S and V717F are shown in the Supplementary Fig. S2.

We identified a 17-year-old boy who was referred to the hospital due to intellectual disability (ID), autism spectrum disorder (ASD) and epilepsy (see Supplementary Data, and Table S1 for clinical description). Trio whole-exome analysis discovered a de novo missense variant c.1927G>C (p.Gly643Arg) in *SHANK2* (NM_012309.5; Fig. 1a, b). This variant was found to be deleterious using predictions programs such as CADD [48]; it is absent from catalogs of human genetic variation (i.e., the GnoMAD database). Using the Genematcher portal [49], we identified one further individual with developmental delay, mild intellectual disability, microcephaly, behavioral abnormalities and attention deficit hyperactivity disorder. Again, a de novo missense variant (c.5399T>G, p.L1800W) in *SHANK2* was found in the patient.

### The p.G643R mutation disrupts binding of Shank2 to GKAP

Shank PDZ domains are type I PDZ domains. PDZ type I ligands carry a C-terminal X − S/T − X − Φ –COO^−^ motif, where X is variable, and Φ hydrophobic. In GKAP, Φ is leucine (L(0) in PDZ terminology), which together with threonine at the −2 position constitutes the main specificity determinants for type I PDZ domains [3, 4]. In addition, an extended sequence N-terminal to the PDZ domain (corresponding to residues 597–628 in Shank2) adds to binding affinity and specificity as it binds to an extended surface of the PDZ ligand [50]. The extended binding site is provided by a second Shank molecule, indicating that a dimer of Shank proteins binds to two GKAP molecules. All 15 C-terminal amino acids of GKAP are required for high affinity interaction with Shank3 [50]. Homology modeling in Fig. 1c shows that also the Shank2/GKAP interaction uses the same extended interface, such that all 15 C-terminal amino acids of GKAP bind to the extended binding site of Shank2.

Upon homology modeling of WT and mutant Shank2 based on the Shank3/GKAP structure [50] we observed that known mutations affect residues in the extended PDZ domain (S610Y) or in regions on the backside of the PDZ domain, facing away from the ligand (N690S, V717F; Fig. 1c). In contrast, G643 is in a central position in the peptide binding groove. G643 is highly conserved in type I PDZ domains (Fig. 1b). In structures of PDZ domain/ligand complexes [51, 52], a glycine at this position anchors a conserved histidine (H698 in Shank2; Fig. 1d) through a hydrogen bond of its carbonyl group with N-1 of H698. N-2 of H698 is involved in hydrogen bonding to the OH-group of Thr at the −2 position of the PDZ ligand, the major determinant of specificity for type I PDZ domain interactions (Fig. 1d). Replacement of Gly643 with Arg introduces a bulky side chain at this position, which clashes with His698 (Fig. 1d). Thus, instead of supporting the correct positioning of H698, Arg643 pushes away His698, thereby distorting the binding site for the GKAP PDZ ligand.

We performed a GST pulldown using the Shank2a isoform containing the extended binding site, and a GST fusion protein containing the complete GKAP PDZ ligand also including the extended binding region in GKAP. We included PDZ domain variants identified in individuals with schizophrenia [23], i.e., p.S610Y (in the extended binding site on Shank2) and p.N690S. Here, the p.G643R variant almost completely disrupts the binding of Shank2 to GKAP through the PDZ domain, whereas the two previously reported mutations only slightly, and non-significantly affected binding (Fig. 1e, f). To obtain more precise data, we also generated His_6_-SUMO tagged fusion proteins encompassing SH3 and PDZ domains of Shank2 (Fig. S1). We measured binding to a synthetic peptide containing the last 15 amino acids of GKAP/SAPAP1, using isothermal titration calorimetry (ITC). Here, the S610Y, N690S [23] and V717F [20] mutants elicited moderate to no reductions in affinity, whereas G643R reduced the affinity more than 70 fold (Figs. 1g, h, S2).

We also performed ITC experiments with a peptide corresponding to the C-terminal PDZ ligand of CIRL1/latrophilin [53]. For this ligand we detected an about 40-fold reduced affinity for the wild type protein, in agreement with previous observations that GKAP is the partner with the highest affinity for Shank PDZ domains. Importantly, binding to G643R mutant Shank2 was not detectable in this assay (Fig. S3), clearly demonstrating that the mutation destroys the core function of the PDZ domain of Shank2.

### The p.L1800W mutation in *SHANK2* interferes with the formation of Shank2 oligomers

**Fig. 2:**
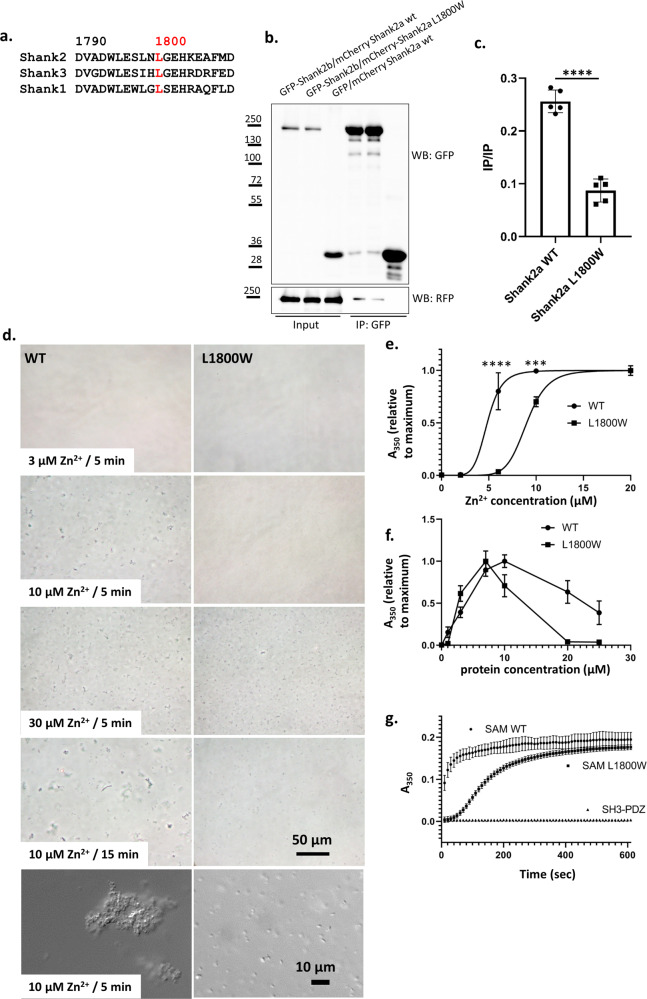
p.L1800W mutation in the SAM domain interferes with Shank2 homo-oligomerization. **a** Alignment of sequences surrounding L1800 (red) in the Shank2-SAM domain with those of other Shank SAM domains. **b** GFP-tagged Shank2b, or GFP control, was coexpressed with mCherry-tagged variants of Shank2a in 293T cells. After cell lysis, GFP-tagged proteins were immunoprecipitated using the GFP-trap matrix. Input and precipitate samples were analysed with GFP- and mRFP-specific antibodies. **c** Quantification of the data shown in (**b**). ****, significantly different, *p* < 0.0001; data from five independent experiments; *t* test. **d** His_6_-SUMO tagged Shank SAM domains (WT and L1800W mutant) at a concentration of 10 µM were treated with various concentrations of Zn^2+^. After 5 min (or 15 min, lower panel), 20 µl drops were spotted on a microscopic slide and photographed through a phase contrast microscope. Higher resolution DIC images were also generated (lower panels). Bars: 50 µm, 10 µm. **e** Samples containing 10 µM SAM domain were treated with different concentrations of Zn^2+^ in wells of a 96-well microtiter plate. Absorbance at 350 nm was measured after 10 min. Data were normalized to the maximum absorbance obtained in each experiment, and analyzed by nonlinear regression using GraphPad Prism software. ****, ***: significantly different, *p* < 0.0001, 0.001, respectively; *t* test. **f** Measurements were performed as in (**d**), however the Zn^2+^ concentration was kept constant while protein concentration was varied as indicated. **g** Protein samples at a concentration of 10 µM were treated with 10 µM Zn^2+^. After the start of the reaction, absorbance was measured at 350 nm every 10 s. Here a His_6_-SUMO fusion protein containing SH3 and PDZ domains of Shank2 was included as a negative control. In (**e–g**), mean ± SD of 4 independent experiments is shown.

Zn^2+^-dependent formation of homo/hetero-oligomers of Shank SAM domains is thought to be fundamental for PSD formation [1, 3, 13]. L1800 is conserved in SAM domains of all three Shank proteins (Fig. 2a). We analysed the capacity of WT and L1800W mutant Shank2 to interact with another Shank2 molecule in a coimmunoprecipitation experiment. Cells coexpressing differently tagged Shank2 proteins (GFP- and mCherry-tagged) were lysed and subjected to immunoprecipitation using the GFP-trap matrix. Here, the p.L1800W variant negatively affects Shank2-Shank2 interactions, as coprecipitation of mCherry-tagged Shank is reduced by the mutation (Fig. 2b, c). It should be noted that this experiment was performed in buffer containing 1 mM EDTA, likely to sequester any Zn^2+^ from cell lysates. However, the Shank2 and Shank3 Zn^2+^ binding sites are of high affinity, and it appears possible that some Zn^2+^ remains attached to SAM domains during the procedure. The residual interaction observed here may also be caused by other domains of Shank2, not only the SAM domain.

Both mutations identified here do not affect binding to Homer, as determined in coexpression/coimmunprecipitation assays, further confirming the specific role of G643 for the PDZ domain, and L1800 for the SAM domain (Fig. S4).

### p.L1800W causes a delayed Zn^2+^-dependent polymerization of SAM domains

We purified His_6_-SUMO-tagged SAM domains (Fig. S1) and established in vitro polymerization assays. WT or L1800W mutant proteins (at 10 µM concentration) were treated with Zn^2+^, and the resulting samples were viewed under a microscope at 5 min after adding Zn^2+^ (Fig. 2d). For the WT, we readily observed large (>5 µm) clusters shortly after adding 10 µM Zn^2+^ whereas at this concentration the L1800W solution remained clear, without cluster formation. At higher concentrations of Zn^2+^ (30 µM), and also at longer incubation times with 10 µM Zn^2+^, the p.L1800W mutant protein also started to form clusters. No clusters were seen at a Zn^2+^ concentration of 3 µM for both variants. Higher magnification pictures at the 10 µM/5 min condition showed huge irregular clusters for the WT SAM domain, whereas multiple, but rather small aggregates appear for the p.L1800W mutant protein (Fig. 2d, lower panel).

For quantitation, we measured absorbance of SAM-domain/Zn^2+^ solutions as a function of time and Zn^2+^ concentration at 350 nm. Here the p.L1800W variant required higher concentrations of Zn^2+^ for efficient cluster formation, with an EC_50_ of 4.8 µM for the WT, and 9 µM for the mutant. The curve for Zn^2+^ concentration dependence was extremely steep for both variants, with no effect seen at the lower concentration of 2 µM Zn^2+^. Nonlinear regression analysis gave Hill coefficients of 6.5 (WT) and 8.2 (L1800W), pointing to high cooperativity of Zn^2+^-dependent aggregation (Fig. 2e).

We also kept Zn^2+^ constant but varied the protein concentration. Absorbance reached a maximum when concentrations of both protein and Zn^2+^ were equal, clearly indicating that a stoichiometry of one Zn^2+^ ion per protein molecule is required for efficient cluster formation (Fig. 2f). A striking difference between WT and mutant was observed in the kinetics of aggregate formation. The WT SAM domain formed aggregates very rapidly, with half of the maximum absorbance already reached at the initial 10 s time point after adding Zn^2+^. In contrast, the mutant showed a lag phase of 30–40 s of no aggregation, before starting an almost exponential increase in absorbance which reaches saturation after about 300–400 s (compared to WT which approaches saturation much earlier). No aggregation was observed for the His_6_-SUMO-SH3-PDZ fusion, used as a negative control here (Fig. 2g). These data indicate that the rapid, cooperative aggregation of SAM domains in the presence of Zn^2+^ is remarkably slowed down in the mutant. Only after an initial, slow polymerization in p.L1800W SAM domains, the mutant can begin with rapid, exponential growth of clusters.

### The p.L1800W variant alters the Zn^2+^ binding site of Shank2

**Fig. 3:**
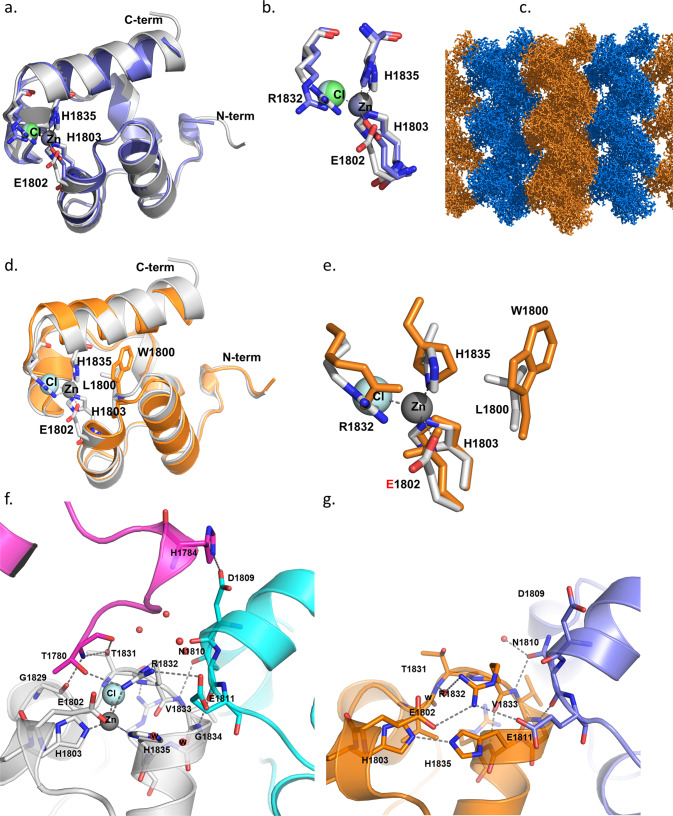
Shank2-SAM binds Zn^2+^ and supports helical sheet formation. **a** Overall fold representation of WT Shank2-SAM domain (colored in gray) aligned with Shank3-SAM domain (colored in blue) [13] (rmsd = 0.614 for superimposing 65 residues); **b** Zoom in representation of the Zn^2+^ cluster observed in both structures. In the Shank2-SAM structure, the Zn^2+^ ion only shows 30% occupancy and its tetrahedral coordination is distorted. The Cl^−^ anion and E1802 are located at a distance longer than expected (2.9 Å and 3.2 Å, respectively), and R1832 is located closer (3.0 Å), establishing an interaction with the Cl^−^ anion (3.2 Å). **c** Crystal packing representation of Shank2-SAM highlighting the observed helical sheets. **d** Overall fold representation of Shank2-SAM-WT domain (gray) aligned with Shank2-SAM-L1800W mutant domain (orange) (rmsd = 0.965 for superimposing 68 residues); **e** Zoom in representation of the Zn^2+^ cluster observed in Shank2-SAM-WT. The introduction of a bulkier side chain (W vs. L) in the mutant SAM domain induces a shift of the C-terminal helix (residues 1831:1849), moving H1835 (one of the Zn^2+^ coordinating residues) 2 Å away from its position in the SAM-WT structure. **f**, **g** Depiction of inter- and intra-fiber interactions in SAM-WT (**f**) and in SAM-L1800W (**g**). SAM-WT monomer is colored in gray, the symmetry related molecules are colored in magenta and cyan, respectively; SAM-L1800W monomer is colored in orange and the symmetry related molecule in blue. Note that there are intrahelical (cyan) and interhelical (magenta) SAM domains in the WT, but only an intrahelical domain for the mutant. Thus, the interhelical domain contact is lost in the mutant.

We obtained crystals of Shank2-SAM domains after cleaving off the His-SUMO tag (Fig. S5); differences in Zn^2+^-dependent aggregation were verified for the isolated domains by dynamic light scattering (DLS; Fig. S6). Upon solving the structure of the WT, we observed high similarity between the Shank2-SAM domain, and the structure of M56E mutant Shank3-SAM domain (see Fig. 3a–c) [13]. Differences were observed in the region of the Zn^2+^ binding site, as the Shank3 crystals had been soaked with Zn^2+^, and the site was fully occupied. Though no Zn^2+^ was added to Shank2 crystals, we observed that about 30% of the Zn^2+^ sites were occupied. Apparently some Zn^2+^ binding had been maintained during purification. Due to the incomplete occupancy, the tetrahedral coordination of the Zn^2+^ ion made by His1803, His1835, a chloride ion and Glu1802 was somewhat distorted and incomplete (Fig. 3b). Nevertheless, the WT SAM domain crystallized in the same space group, showing the same crystal packing as reported for Shank3. Importantly, this packing arrangement allowed for the formation of helical polymers of SAM domains, and the side-by-side alignment of these helices, leading to larger sheets of polymerized SAM domains (Fig. 3c). As reported by [13], contacts via the intrahelical interface can be distinguished from the additional inter-polymer interface. Both interfaces are supported by the Zn^2+^ binding site which is localized at the contact site between these two interfaces. In the electron density map (Fig. S7), the N-terminal Thr residue (T1780) of a neighboring SAM domain is visible; this is in fact a SAM domain from the adjacent fiber, representing an interhelical contact (see below).

On the other hand, we could not readily generate crystals from the L1800W mutant SAM domain. After one round of optimization, crystals were obtained that diffracted to 1.95 Å. Structural comparison between the crystals structures of WT and of the L1800W mutant SAM domains shows that the bulky side chain of the Trp induces a shift of the C-terminal helix, locating it further away from the Zn^2+^ cluster (Fig. 3d, e). This shift places H1835 about 2.0 Å away from the position observed for SAM WT. Furthermore, H1835 adopts a different conformation for its side chain, as well as the side chain of R1832. As a result, the Zn^2+^ binding site is altered or possibly destroyed. It will be interesting to structurally analyse larger C-terminal fragments of Shank2, to determine whether other parts of the protein affect polymerization. However, we found larger fragments difficult to purify and crystallize (not shown).

The crystal packing of the mutant SAM domain was remarkably different from the WT, as no helical sheets similar to those seen in Fig. 3c for the WT were obtained with the mutant. A closer inspection of the interactions of SAM domains within the crystals showed that the WT domain established several interactions with the neighboring molecules, which are responsible for the formation of helical fibers (Fig. 3f; see also Table S3). In addition, we observed interactions to SAM domains in the neighboring helix. These are needed for the side-by-side stacking of helices, which is a hallmark of Shank SAM domains [13]. While the intrahelical contacts are partially maintained in the L1800W mutant, the interhelical contacts are completely lost as no neighboring molecule is observed at the appropriate position in the crystal (Fig. 3g and Table S3).

### *SHANK2* mutations interfere with postsynaptic targeting

**Fig. 4:**
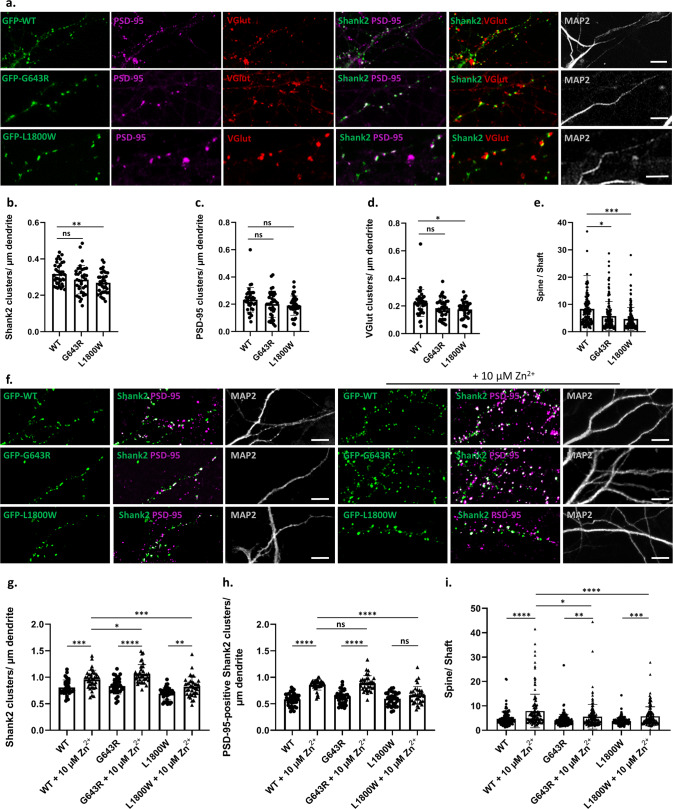
Both mutant variants of Shank2 fail to properly reach postsynaptic sites. **a** Primary cultured rat hippocampal neurons were co-transfected with an shRNA vector against the rat Shank2 mRNA, and an expression vector coding for GFP-tagged human Shank2 (WT or mutant, as indicated). Cells were stained with antibodies against the dendritic marker MAP2, the postsynaptic marker PSD-95, and the presynaptic marker VGlut. **a**, **f** Cells were analysed by confocal microscopy. Scale bar: 5 µm. **b–d**, **g–h** Quantitative analysis of at least 36 dendritic branches of 12–15 neurons obtained from three independent experiments. **b** The number of Shank2-positive clusters is decreased significantly upon expression of the L1800W mutant. **c** The number of PSD-95-positive clusters showed no difference among the three conditions. **d** The number of VGlut-positive clusters is reduced in neurons expressing the L1800W variant of Shank2. **e**, **i** Quantitative analysis of 140 clusters along dendrites of 12-15 neurons obtained from three independent experiments. The ratio of intensity of a GFP-positive dendritic cluster in relation to the intensity in the adjacent dendritic shaft was determined. **e** The spine/shaft signal intensity ratio was significantly reduced in both mutants when compared to the WT. **f–h** Cells were treated with a concentration of 10 µM Zn^2+^. **g** The number of Shank2 clusters is increased for all variants after Zn^2+^ treatment. **h** The number of PSD-95-positive Shank2 clusters is increased for WT and G643R variant after Zn^2+^ treatment, but not for the L1800W mutant. **i** The spine/shaft signal intensity ratio was significantly increased for all three conditions after Zn^2+^ treatment. *, **, ***, ****: significantly different, *p* < 0.05, 0.01, 0.001, 0.0001 respectively; two-way ANOVA, followed by both Sidak’s and Dunnett’s multiple comparison test.

We investigated the effect of both mutations on the postsynaptic localization of Shank2 in primary cultured hippocampal neurons. The endogenous rat Shank2 was knocked down using Shank2 shRNA (ShShank2) constructs described by [29]. This approach targets almost exclusively excitatory, glutamatergic neurons, as only a low percentage (about 6%) of neurons in this type of culture is inhibitory [54]. Efficient knockdown was verified on a Western Blot level in transfected 293T cells, and in primary cultured neurons by immunocytochemistry (Fig. S8). The knockdown construct was coexpressed with GFP-tagged human *SHANK2* variants, which are not susceptible to the shRNA due to sequence divergence of the shRNA target (Fig. S8). Transfected neurons were initially analysed by confocal microscopy (Fig. 4a). We observed that neuronal morphology (i.e., the number of primary dendrites and the length of dendritic spines) were not significantly altered under any of the conditions tested here (Fig. S9). We found that the number of dendritic GFP-Shank2-positive clusters per dendrite length was reduced by the L1800W SAM domain mutation, but not by the G643R PDZ mutation (Fig. 4b). Interestingly, both overexpressed variants of Shank2 showed no effect on the density of PSD-95 clusters along the dendrite (Fig. 4c), whereas the L1800W variant showed a decreased density of presynaptic clusters of VGlut which were positive for Shank2 (Fig. 4d). Importantly, when we measured the ratio of cluster/spine fluorescence intensity versus the intensity in the adjacent dendritic shaft, we saw a significant reduction in this ratio for both mutants when compared to the wild type (Fig. 4e). Our data indicate that both the PDZ domain and the C-terminal SAM domain contribute to the targeting of Shank2 to the postsynaptic sites and mutations in both functional domains may affect the proper targeting of this protein to postsynaptic sites.

As Shank3 can perform many of the same interactions and functions as Shank2, Shank3 might in some way compensate for non-functional Shank2. We analysed cells expressing WT and mutant protein for changes in the postsynaptic targeting of the endogenous Shank3 protein. Indeed, we observed an increase in the number of dendritic Shank3 clusters in neurons expressing G643R-Shank2 (Fig. S10).

We investigated whether the deficits of Shank2 mutants in forming postsynaptic clusters can be rescued by a higher Zn^2+^ concentration. Increasing Zn^2+^ concentration for a short period of time (1 h, additional 10 µM Zn^2+^) had no apparent effect on Shank2 clustering (data not shown). In contrast, long term treatment of cultures with additional Zn^2+^ significantly increased the number of Shank2 clusters for WT Shank2, as well as the number of PSD-95-positive Shank2 clusters and the spine to shaft ratio. For the G643R mutant, similar changes were observed in the number of clusters, and, to a lesser extent, in the spine/shaft ratio. For the L1800W variant, we observed only a rather weak effect of Zn^2+^ treatment for all three parameters, with no significant change in the number of PSD-95 positive clusters (Fig. 4f–i).

### *SHANK2* mutations interfere with the formation of postsynaptic nanoclusters

**Fig. 5:**
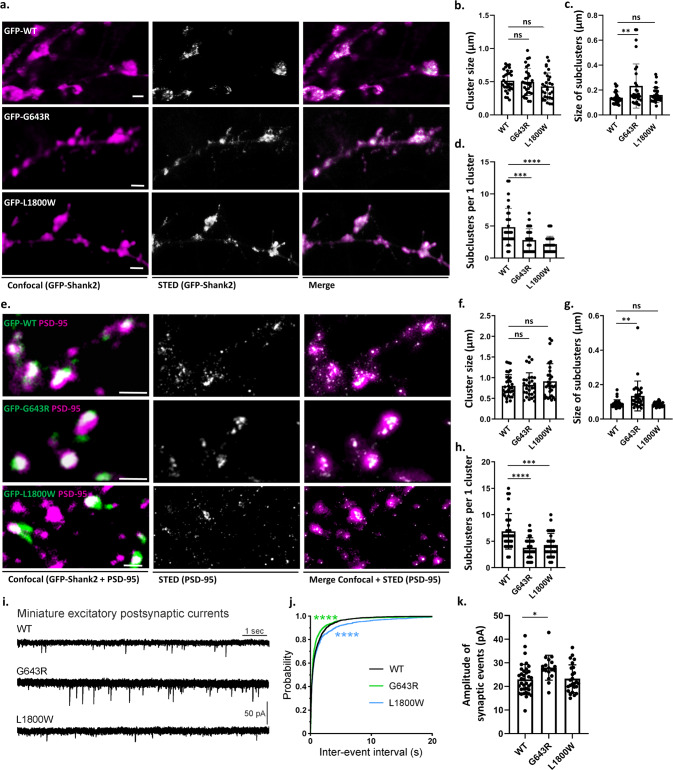
Mutations in *SHANK2* alter the nanoscale organization of both Shank2 and PSD-95 in the postsynaptic density. Rat hippocampal neurons cotransfected with GFP-tagged human Shank2 constructs and a shShank2 vector, were stained with antibodies against GFP as well as PSD-95. Confocal and 2D STED imaging was performed on GFP-Shank2 clusters (**a**) or on PSD-95 clusters colocalising with Shank2 (**e**; areas of colocalisation are indicated in white, left panel). Single clusters of Shank2/PSD-95 observed in confocal mode were resolved into subclusters in super-resolution mode. Scale bar: 1 µm. Quantitative analysis was performed on 30 clusters from 10 neurons per each condition, obtained from three independent experiments. **b** The size of clusters in the confocal mode did not show any difference between WT Shank2 and the two mutant variants. **c** The size of Shank2 subclusters is significantly increased when expressing the G643R mutant compared to WT and L1800W. **d** Number of subclusters was significantly higher in clusters positive for WT Shank2 compared to both mutant variants. **f** The PSD-95 cluster size (confocal mode) was not significantly altered among the variants. **g** The size of PSD-95 subclusters was increased when expressing the G643R mutant compared to WT and L1800W. **h** Number of PSD-95 subclusters per cluster was significantly reduced for both mutant variants. **, ***, ****: significantly different, *p* < 0.01, 0.001, 0.0001 respectively; Kruskal–Wallis test, followed by Dunn’s multiple comparison test. **i** Miniature excitatory postsynaptic currents (mEPSCs) recorded in primary cultured hippocampal neurons cotransfected with shShank2 vector and human Shank2 construct. **j** Cumulative probability of mEPSC inter-event intervals was significantly altered for both variants. ****: *p* < 0.0001; Kolmogorov–Smirnov test. **k** The mean amplitude of mEPSCs was significantly larger in p.G643R variant cells compared to wild type cells. *p < 0.05; one-way ANOVA with Dunnett’s multiple comparison test.

The postsynaptic localization of scaffold proteins is not uniform, but occurs in so-called nanoclusters; several of these ∼80 nm structures may be distinguished in a single spine head [14, 15]. Determinants which are responsible for targeting of Shank or other postsynaptic proteins to these nanoclusters are unknown. We combined confocal microscopy and 2D STED imaging on Shank2-positive clusters from transfected neurons (Fig. 5a). Clusters were identified in the confocal mode, and then further resolved by STED microscopy. We routinely observed multiple subclusters in the STED images. Although the size of clusters was similar in all three conditions (Fig. 5b), for the p.G643R variant, we observed that the size of subclusters is increased in comparison to subclusters containing the WT Shank2 (Fig. 5c). For both mutants, the number of the subclusters was significantly decreased (Fig. 5d), suggesting that the compaction of Shank2 protein into nanoclusters is reduced by the mutations (Fig. 5c, d).

In a second set of experiments, we focused our analysis on nanoclusters of PSD-95, which is indirectly associated with Shank2 via GKAP. In GFP-Shank2-positive dendritic spines, we observed that PSD-95 nanoclusters were altered in the same manner as the Shank2 clusters, as the size of nanoclusters was increased for neurons expressing the p.G643R variant, and the number of nanoclusters was reduced for both variants when compared to the wild type (Fig. 5e–h). On the other hand, STED microscopy of presynaptic VGlut clusters did not reveal any differences between cultures expressing the different Shank2 variants (Fig. S11).

To assess the effects of Shank2 mutants on synaptic function, we recorded miniature excitatory postsynaptic currents (mEPSCs) in primary cultured hippocampal neurons. In neurons expressing the p.L1800W variant (*n* = 25), the inter-event interval of mEPSCs was significantly increased compared to wild type (*n* = 36), consistent with the reduction in the number of functional synaptic sites, as observed in Fig. 4b, d. The inter-event interval of mEPSCs was slightly but significantly decreased in neurons expressing the p.G643R variant (*n* = 18) (Fig. 5i, j). In addition, the mean mEPSC amplitude was significantly larger for the p.G643R variant but unaltered for the p.L1800W variant (Fig. 5k).

## Discussion

Whereas many mutations in *SHANK* genes have been described in individuals with neurodevelopmental disorders [16], little is known about the functional effects of missense mutations on the molecular properties of the encoded proteins. We describe two patients with *de novo* missense variants in *SHANK2* affected by a severe neurodevelopmental disorder, comprising moderate intellectual disability, delayed acquisition of motor and language skills, but also seizures (patient 1) and microcephaly (patient 2). Patients appear to be more severely affected than those carrying heterozygous loss-of-function variants, occurring e.g., trough frameshift or early stop mutations [55]. The variants alter two domains which are of central relevance for Shank protein function, i.e., the PDZ and SAM domains [3, 13]. To our knowledge these are the first de novo variants in these two domains which show a clear functional deficit for either domain, detectable in structural, biochemical as well as cell biological assays.

Three additional *SHANK2* variants in the PDZ domain had been observed in patients (p.V717F in an autism case, p.S610Y and p.N690S in schizophrenia patients [20, 23]). As the effects of these variants on interaction with GKAP had not been analysed, we included them here in our binding assays. GKAP is believed to be the most relevant partner of Shank PDZ domains, as it uses an extended interface with Shank proteins for high affinity interaction [50]. The p.G643R variant almost eliminates binding of GKAP to the PDZ domain of Shank2. G643 is highly conserved within type I PDZ domains, and our molecular modeling as well as published 3D structures suggest that the large Arg residue at position 643 destroys one of the major hallmarks of type I PDZ domain interactions, i.e., the selectivity for the obligatory Ser/Thr residue at the (-2) position of the ligand. For the S610Y and V717F variants, a moderate reduction in binding affinity was observed, consistent with the localization of S610 and V717 outside the immediate ligand binding site (Fig. 1). The reduced affinity of the S610Y variant supports the relevance of the extended PDZ domain. It should be noted that the phenotype of the patient reported here is more severe, with a more general neurodevelopmental delay compared to the autism (V717F) and schizophrenia (S610Y) phenotypes caused by the other mutations [20, 23]. The lack of effect of the N690S variant questions its pathogenicity.

The p.L1800W variant is the first missense variant affecting the function of Shank SAM domains. Polymerization into helical structures, as well as the side-by-side alignment of these helices into sheet-like structures as observed for Shank3 is dependent on the presence of Zn^2+^ [13]. By varying the concentration of Zn^2+^ in microscopic and photometric aggregation assays, we observed here that aggregation does not increase in a linear manner with increasing Zn^2+^ concentrations, but instead shows an S-shaped curve reminiscent of the oxygen binding curve of hemoglobin. Hill coefficients between 6 and 8 clearly indicate that Shank2-SAM domain aggregation is a highly cooperative process. This may have important therapeutic consequences, as very small increases in the available postsynaptic Zn^2+^ concentration (e.g., through increased dietary uptake) may have a profound effect on the assembly of postsynaptic Shank clusters (as observed recently, [56]). We found that this cooperative process is severely delayed by the p.L1800W mutation. Our structural analysis of WT and mutant Shank2-SAM domains provides a molecular explanation for these findings. Replacement of L1800 with a large, aromatic residue leads to an outside movement of the carboxy-terminal helix, thereby detaching His1835 from the tetrahedral coordination sphere of the Zn^2+^ ion. As a result, Zn^2+^ affinity is reduced to the effect that no Zn^2+^ is found in the mutant protein, whereas WT SAM domain keeps partially attached to its bound Zn^2+^ during the purification and crystallization procedure. Though Zn^2+^ binding is apparently still possible, the conformational changes disrupt several interactions with other SAM domains in the polymeric assembly process. This is especially true for the contact between helical fibers of SAM domains, but not within these helices; thus, the mutation affects a Shank-specific feature of SAM domains, the ability to form larger sheets through side-by side stacking of the helices described by [13] and also observed here for WT Shank2. This deficit in the two-dimensional packing of SAM domains is likely to be responsible for the delayed polymerization which was observed in our in vitro aggregation assays (see Fig. S12 for a model).

Functional studies were performed in primary cultured hippocampal neurons, which endogenously express all three Shank isoforms. We expressed GFP-tagged Shank2 variants together with a knockdown plasmid for the endogenous protein, to avoid “Shank overload”. While it is difficult to precisely measure the total amount of Shank2, we think that this creates a homozygous situation for the expressed Shank2 mutants. We felt that this is necessary to observe a robust phenotype. In fact, functional studies in model organisms (e.g., in Shank ko mice) usually rely on a homozygous situation in order to detect a phenotype [57–59], despite the fact that *SHANK* mutations in human patients are always heterozygous. In two recent studies, even double knockout mouse lines (deleting a total of four *Shank* allels) have been analysed to more clearly assess the function of Shank proteins in synaptic function and autism pathology [60, 61].

Upon expression in neurons, both mutations reduced the efficiency of targeting of the Shank2 protein to postsynaptic clusters. Previous work has led to divergent results regarding the role of Shank domains for synaptic targeting. Thus, targeting of Shank1 depends on the PDZ domain [62], and therefore, on the interaction with GKAP, whereas Shank2 and Shank3 require an intact C-terminal synaptic targeting motif which includes a functional SAM domain [63]. Our work suggests an intermediate view for Shank2, as both intact PDZ and SAM domains contribute to efficient targeting to postsynaptic sites. Differences between our experiments and previous studies may originate from the use of CMV-promoter based constructs which drive high levels of expression. We have used here expression constructs based on the EF1α promoter, which may bring about more physiological expression levels.

Addition of Zn^2+^ to neuronal cultures increases postsynaptic clustering of Shank2 and Shank3 [64]. We estimate the physiological total Zn^2+^ concentration in neuronal cultures to be in the lower µM range, whereas the free Zn^2+^ concentration is believed to be highly variable. By adding further Zn^2+^, free Zn^2+^ levels are likely to rise. In our hands, endogenous (e.g., Fig. S10) and overexpressed Shank proteins are efficiently clustered and targeted to postsynaptic sites [28, 45], suggesting that this concentration of Zn^2+^ is sufficient for the amount of Shank present in dendrites. However, by adding 10 µM Zn^2+^ to cultures for 3.5 days, we observed increased formation of PSD-95 positive, postsynaptic clusters for WT and G643R mutant Shank2, but not for the L1800W mutant. Also the increase in spine targeting of the protein was less pronounced for the SAM domain mutant, further confirming that Zn^2+^ dependent polymerization of the Shank2-SAM domain mediates the effect of Zn^2+^ on synapse formation. This is in agreement with in vivo data, showing that limited availability of Zn^2+^ during mouse development impairs targeting of Shank proteins to synaptic sites, and is associated with the development of autism-like symptoms [65, 66].

Reduced targeting to postsynaptic sites coincides for both mutants with changes in the formation of postsynaptic nanoclusters which are responsible for the tight spatial association of the presynaptic neurotransmitter release machinery with postsynaptic AMPA receptors [14, 15]. So far it is unknown how formation and the correct positioning of nanoclusters is achieved on a molecular level. Recent in vitro work has shown that interactions between scaffolds PSD-95, GKAP, Shank and Homer drive a liquid-liquid phase transition, which is likely to be involved in postsynaptic clustering and nanocluster formation [67]. The PDZ-mediated GKAP-Shank interaction, as well as the SAM domain mediated polymerization of Shank proteins feature prominently in this process. It is likely that these interactions first serve to bring the postsynaptic proteins into close proximity; in a next step, the large intrinsically disordered segments of all three Shank proteins are likely to perform the phase transition which then creates the really “dense” nanoclusters observed by super-resolution microscopy. Thus, the mutations identified here interfere with the compaction of postsynaptic clusters, by affecting two of the most important interactions mediated by Shank proteins. We would assume that GKAP nanoclusters are affected in a similar manner; however, we are currently not aware of a suitable antibody to analyse this. Importantly, we show that the changes in nanoclusters extend throughout the PSD, as PSD-95 clusters are also affected in a similar manner as Shank2 clusters by both mutations (Fig. 5). This confirms that Shank multimerization, and formation of the Shank/GKAP/PSD-95 complex, are central aspects of nanocluster formation which are severely disrupted by the mutations found in our patients.

Both mutations analysed here are associated with altered synaptic transmission (Fig. 5). mEPSCs are less frequent for the L1800W mutant, in agreement with the reduced number of functional synapses (VGlut-positive Shank2 clusters) observed upon expression of this variant. In case of the PDZ domain mutant, in fact we see a slight increase in the number of mEPSCs, coinciding with an increase in signal amplitude. These data indicate that there is no clear correlation between nanocluster formation and strength of postsynaptic currents. It should be noted here that we actually observed larger nanoclusters in neurons expressing this variant (Fig. 5), and that there was a compensatory response by Shank3 in these neurons (Fig. S10). Postsynaptic content of Shank3 was also increased in the Shank2 ko mice, indicating that Shank3 may take over as interaction partner for the GKAP C-terminus in the absence of Shank2; no increase for Shank1 was observed in these mice [58].

We believe that, e.g., upon introduction into the mouse genome, these mutations will provide an important opportunity to study the relevance of nanocluster formation for synaptic signaling and synaptic plasticity. These aspects cannot easily be addressed in a cultured neuron system. Furthermore, it may be interesting to vary the Zn^2+^ concentration before or during electrophysiological measurements.

As the patients described here appear to be more severely affected than those carrying heterozygous loss-of-function variants [55], our data suggest that nanocluster compaction is indeed a central aspect of Shank2 function. Furthermore, our data suggest a dominant pathomechanism, where the mutant Shank2 allel is dominant over the WT allel, and possibly also over the other two SHANK genes which send their gene products to the same postsynaptic sites. Our experience with other genes causing neurodevelopmental disorders shows that often missense variants cause a more severe phenotype than loss-of-function variants. This happens when variants do not disrupt folding and overall abundance of the gene product, but rather interfere with one specific and important molecular interaction while leaving others intact [68–70]. For Shank2 variants described here, other interactions outside PDZ (for p.G643R) and SAM (for p.L1800W) domains are not affected; targeting of the mutant protein to postsynaptic sites is reduced but not abolished. As Shank proteins in the PSD are part of a dense network of protein interactions, this suggests that this network is disrupted at one particular position (the PDZ-GKAP contact, or the interface between two helices of SAM domains), causing a disrupted molecular pattern. Due to the tight incorporation of Shank2 (mutant or WT) into the PSD complex, this cannot be fixed by the presence of other intact Shank proteins, such as Shank3 which is mostly equivalent to Shank2 in its protein interactions, and which is increased in abundance at postsynaptic sites upon introducing a Shank2 mutation (Fig. S10). Cotransfection of WT and mutant Shank2 (e.g., with two different tags) upon a knockdown background would be the ideal experiment to experimentally show that mutant Shank2 is also dominant over WT Shank2. However, we find this type of triple transfection (shRNA + two expression vectors) of neurons difficult to perform in a controlled manner, as we cannot determine whether equal amounts of both proteins are expressed. Due to the low percentage of transfected cells, our cultured neurons are not susceptible to Western Blot analysis, which would be the proper way to do this.

In summary, our data for the first time show that PDZ- and SAM-domain mediated interactions of Shank proteins are required for shaping and compaction of postsynaptic nanoclusters, and that this is relevant for the healthy development of the human nervous system.

## Supplementary information

Supplemental Material

## Data Availability

Macromolecular structures have been submitted to the RCSB Protein data bank (https://www.rcsb.org/) under accession numbers 8ATJ (SAM domain, WT) and 8B10 (SAM domain, L1800W). All other data are available in the main manuscript and in the Supplementary Material.
