# Supplementary material for: Structural deficits in key domains of Shank2 lead to alterations in postsynaptic nanoclusters and to a neurodevelopmental disorder in humans
Source: Mol Psychiatry. 2022 Nov 30;29(6):1683–97. doi: 10.1038/s41380-022-01882-3 (PMC11371640; doi:10.1038/s41380-022-01882-3)
Supplement: Supplementary file 1 — Supplemental Material [file 41380_2022_1882_MOESM1_ESM.docx]

**Supplemental data; Hassani Nia et al, 2022**

**Clinical description, patient 1.**

The proband is a 17-year-old boy, who was referred to our hospital at the age of 16 years due to intellectual disability (ID), autism spectrum disorder (ASD) and epilepsy. He was the first child of nonconsanguineous parents, who was born at 35 weeks’ gestational age with normal birth measurements. Family history is notable for a maternal uncle with ID and paternal cousin with mild developmental delay. The neonatal period was complicated by pyloric stenosis requiring surgical intervention at the age of 5 months. Motoric milestones were delayed: he started walking at the age of 30 months. Language development was delayed as well: he started talking in 2-word sentences at the age of 5 years. In toddler age a lack of social interaction along with restricted, stereotypic, and ritualized patterns of interests and behavior became evident. According to a suspected inner ear hearing impairment he received hearing devices at the age of 2.5 years. At the age of 10 years absence epilepsy occurred with sporadic grand mal seizures in the further course. Under anticonvulsive medication he became seizure-free. Brain MRI showed normal result at the age of 17 years. In adolescence age, the boy was diagnosed with Scheuermann’s disease and he underwent surgery to correct his *genua valga*. Previous genetic testing including chromosome analysis, array CGH analysis, fragile X testing as well as panel analysis for X-linked intellectual disability (XLID) yielded normal results. Trio whole exome analysis (WES) discovered a *de novo* missense variant c.1927G>C [p.(Gly643Arg)] in *SHANK2* (NM_012309.5).

**Supplementary Table 1: clinical features of patient 1**

|  |  | Patient 1 |
| --- | --- | --- |
| General Information | Gender | Male |
|  | Ethnicity | German |
|  | Age at last examination | 17 years |
| Patient Genotype/  Molecular diagnosis | Gene | *SHANK2* |
|  | Chromosome position | 11q13.3-q13.4 |
|  | Reference sequence | NM_012309.5 |
|  | nucleotide/amino acid change | c.1927G>C [p.(Gly643Arg)] |
|  | hg build | hg19; Chr.11:g.70507710 |
|  | Inheritance | *De novo* |
|  | *In silico* predictions and catalogs of genetic variation in humans | CADD: 32; REVEL: 0.347; M-CAP: 0.157  Polyphen: 1.000  SIFT: deleterious  Provean: damaging  GnoMAD: absent |
| Family History | Consanguineous parents | No |
|  | Number of siblings | Two half siblings |
|  | Similarly affected siblings | None |
|  | Congenital abnormalities in family members | None |
|  | Intellectual disability in family members | Uncle w/ ID; Cousin w/ mild DD |
|  | Other anomalies in family members | None |
| Prenatal and neonatal history | Prenatal structural anomalies (seen by ultrasound or fetal MRI) | Oligohydramnios |
|  | Gestational age at birth | 34+0 |
|  | Mode of birth | Vaginal delivery |
|  | APGAR at 1/5/10 min | 7/8/9 |
|  | birth length in cm (z-score) | 51cm (0.6z) |
|  | birth weight in g (z-score) | 2880g (0.6z) |
|  | OFC at birth in cm (z-score) | 35 (1.2z) |
|  | Congenital abnormalities | None |
|  | Other neonatal problems | Pyloric stenosis |
| Last exam. | Age | 17 |
|  | Height in cm (z score) | 180.1 (0.1 z) |
|  | Weight in kg (z score) | 68 (-0.1 z) |
|  | OFC in cm (z score) | 58.2 (1.1 z) |
| Development | Motor delay | Yes |
|  | Age at walking unaided | 30 months |
|  | Speech delay | Yes |
|  | Age at first words | unknown |
|  | Current number of words | unknown |
|  | Intellectual disability | Yes |
|  | Degree of intellectual disability | Moderate |
|  | Course of disease | Slow |
| Neurologic and psychiatric features | Brain abnormalities (MRI or CT) | None |
|  | Seizures | Absence epilepsy and Grand-mal seizure |
|  | Abnormal EEG | Irregular theta paroxysms, spike-wave paroxysms |
|  | Behavioral problems | ASD |
|  | Sleep disturbance | Yes |
| Dysmorphic features | Face | Triangular face |
|  | Other | none |
| Other findings | Hearing impairment | Suspected inner ear hearing loss |
|  | Visual impairment | None |
|  | Abnormality of the heart | None |
|  | Respiratory Abnormalities | None |
|  | gastrointestinal abnormalities | Pyloric stenosis |
|  | Urogenital Abnormalities | None |
|  | skin / hair / nail abnormalities | No |
|  | Musculoskeletal Abnormalities | Scheuermann disease, *genu valgum* |
|  | Muscular hypotonia | Yes |
|  | Endocrine abnormalities | No |
|  | Immunological abnormalities | No |
|  | Anomalies of metabolism | None |
|  | Other abnormalities | Chronic headache |
| Previous genetic testing | Gene panels | XLID Panel normal, GJB2 (Connexin 26) analysis normal |
|  | Array-CGH | Normal |
|  | Fragile X | Normal |
|  | Karyotype | 46, XY |

**Supplementary Table 2.** Data and statistics for detemrination of X-ray structures

| **PROTEIN** | **SHANK2-SAM-WT** | **SHANK2-SAM-L1800W** |
| --- | --- | --- |
| **Data collection** |  |  |
| X-ray diffraction source | PETRAIII, P14 | PETRAIII, P14 |
| Wavelength (Å) | 0.9762 | 0.9762 |
| Resolution range (Å) | 49.8 – 2.1 (2.16-2.10) | 65.77 - 1.95 (2.02 - 1.95) |
| Space group | P 6_5_ | C222_1_ |
| Unit cell (Å)/ (^o^) | 57.49 57.49 48.0 90.0 90.0 120.0 | 58.94 135.282 131.548 90 90 90 |
| Reflections total | 102906 (10140) | 518588 (44310) |
| Unique reflections | 5201 (516) | 38501 (3618) |
| Multiplicity | 19.8 (19.7) | 13.5 (12.2) |
| Completeness (%) | 99.92 (100.00) | 99.32 (95.64) |
| <I/σ(I)> | 10.07 (1.61) | 20.04 (1.80) |
| R-merge | 0.2587 (1.42) | 0.071 (1.32) |
| R-pim | 0.05916 (0.3268) | 0.02018 (0.3855) |
| CC_1/2_ | 0.996 (0.568) | 0.999 (0.774) |
| **Refinement** |  |  |
| No. of all reflections | 5199 (516) | 38480 (3618) |
| No. of free reflections | 253 (24) | 1945 (167) |
| R factor | 0.195 | 0.197 |
| R free | 0.228 | 0.256 |
| *Number of non-hydrogen atoms* |  |  |
| macromolecules | 569 | 3455 |
| ligands | 5 | 16 |
| solvent | 52 | 241 |
| Protein residues | 70 | 412 |
| RMS(bonds) (Å) | 0.015 | 0.017 |
| RMS(angles) (^o^) | 1.82 | 2.03 |
| Ramachandran favored (%) | 95.59 | 99.50 |
| Ramachandran allowed (%) | 4.41 | 0.50 |
| Ramachandran outliers (%) | 0.00 | 0.00 |
| Rotamer outliers (%) | 1.59 | 2.89 |
| Clashscore | 4.39 | 3.76 |
| *<B> (Å^2^)* |  |  |
| Overall | 41.4 | 38.99 |
| macromolecules | 41.0 | 38.18 |
| ligands | 37.9 | 36.44 |
| solvent | 45.6 | 50.12 |

**Supplementary** **Table 3. Intrahelical and interhelical interactions between SAM domain monomers observed in the two crystal structures**.

| **Intra-helical interactions (H-Bonds and Salt bridges)** | | | | |
| --- | --- | --- | --- | --- |
| **Monomer1** | **Monomer2** | **Monomer3** | **Length (Å)** |  |
| N1810 (OD1) | (N) V1833 |  | 3.1 | Found in SAM WT and L1800W mutant |
| N1810 (O) | (N) G1834 |  | 2.9 |  |
| R1841(NH1) | (O)S1815 |  | 3.2 | Found only in  SAM-WT |
| R1841(NH1) | (OG)S1815 |  | 3.2 |  |
| R1841(NH2) | (OG)S1815 |  | 3.2 |  |
| K1822 (NZ) | (OD1)D1827 |  | 3.8 |  |
| K1822(NZ) | (OD2)D1827 |  | 2.5 |  |
| K1822(NZ) | (OE2)E1823 |  | 2.8 |  |
| **R1832(NH1)** | **(OE2)E1811** |  | **3.4** |  |
| R1836(NH2) | (OD2)D1827 |  | 3.6 |  |
| **Inter- helical interactions** | | | | |
| **Monomer1** | **Monomer2** | **Monomer3** | **Length (Å)** |  |
| **R1832(NH2)** |  | **(OG1)T1780** | **3.0** | Found only in  SAM-WT.  L1800W does not have this interface. The crystal packing does not show a monomer3 at this position. |
| T1831(OG1) |  | (O)T1780 | 3.4 |  |
| N1799(ND2) |  | (O)Q1845 | 3.2 |  |
| T1831(OG1) |  | (N)1T1780 | 3.0 |  |
| G1829(O) |  | (N)T1780 | 2.8 |  |
| N1799(OD1) |  | (NE2)Q1845 | 3.0 |  |
|  | D1809(OD1) | (NE2)H1784 | 2.8 |  |
|  | D1809(OD1) | (NE2)H1784 | 3.1 |  |

Interactions of R1832 have been highlighted as it participates in both intra- and interhelical contacts. Color coding corresponds to monomers shown in Fig. 3f,g. Letters in parentheses denominate the element (first letter) and the position in the side chain (second letter, with D,Z, E, G refering to delta, zeta, epsilon and gamma positions).


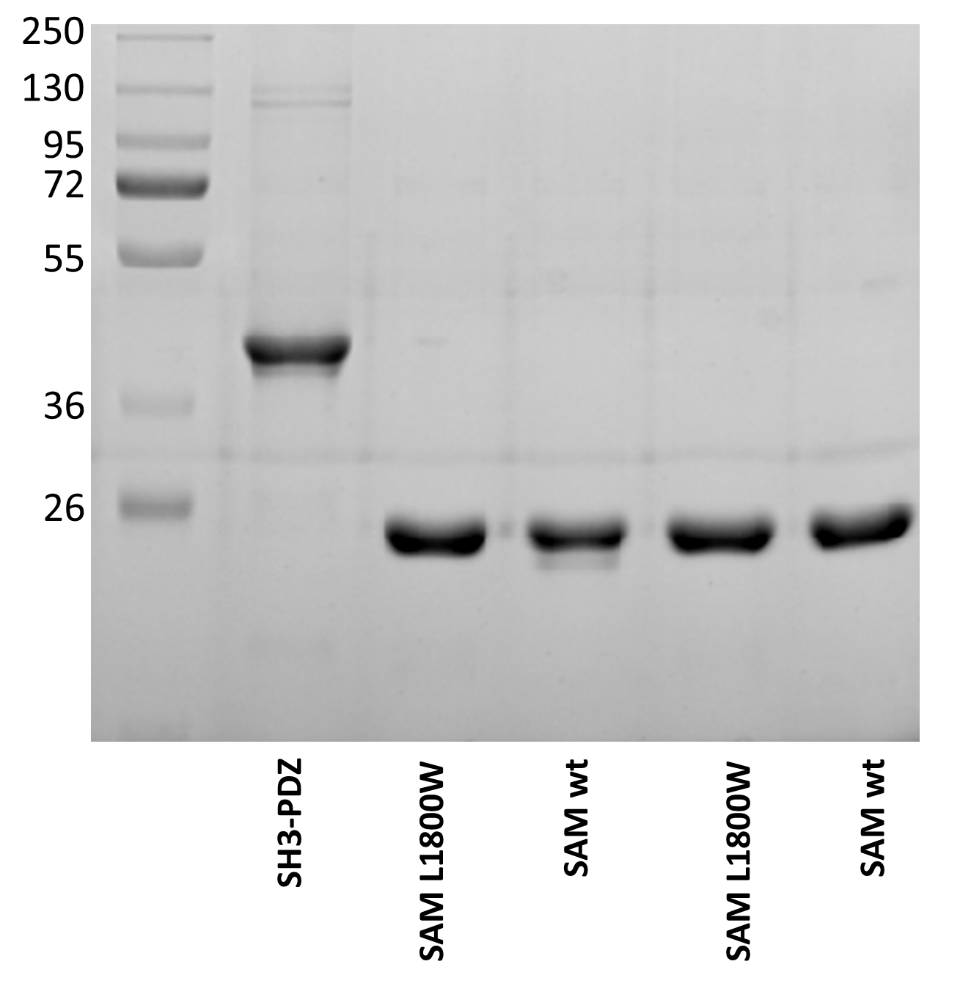


**Supplemental Figure S1. Purification of Shank2 fusion proteins**. Samples of purified His_6_-SUMO-tagged protein fragments of Shank2 were analysed by SDS-PAGE, followed by staining with Coomassie brilliant Blue. Calculated molecular weights are 36.5 kDa (SH3-PDZ; residues Arg520 – Asp727) and 21.5 kDa (SAM; residues Thr1780 – Arg1849).


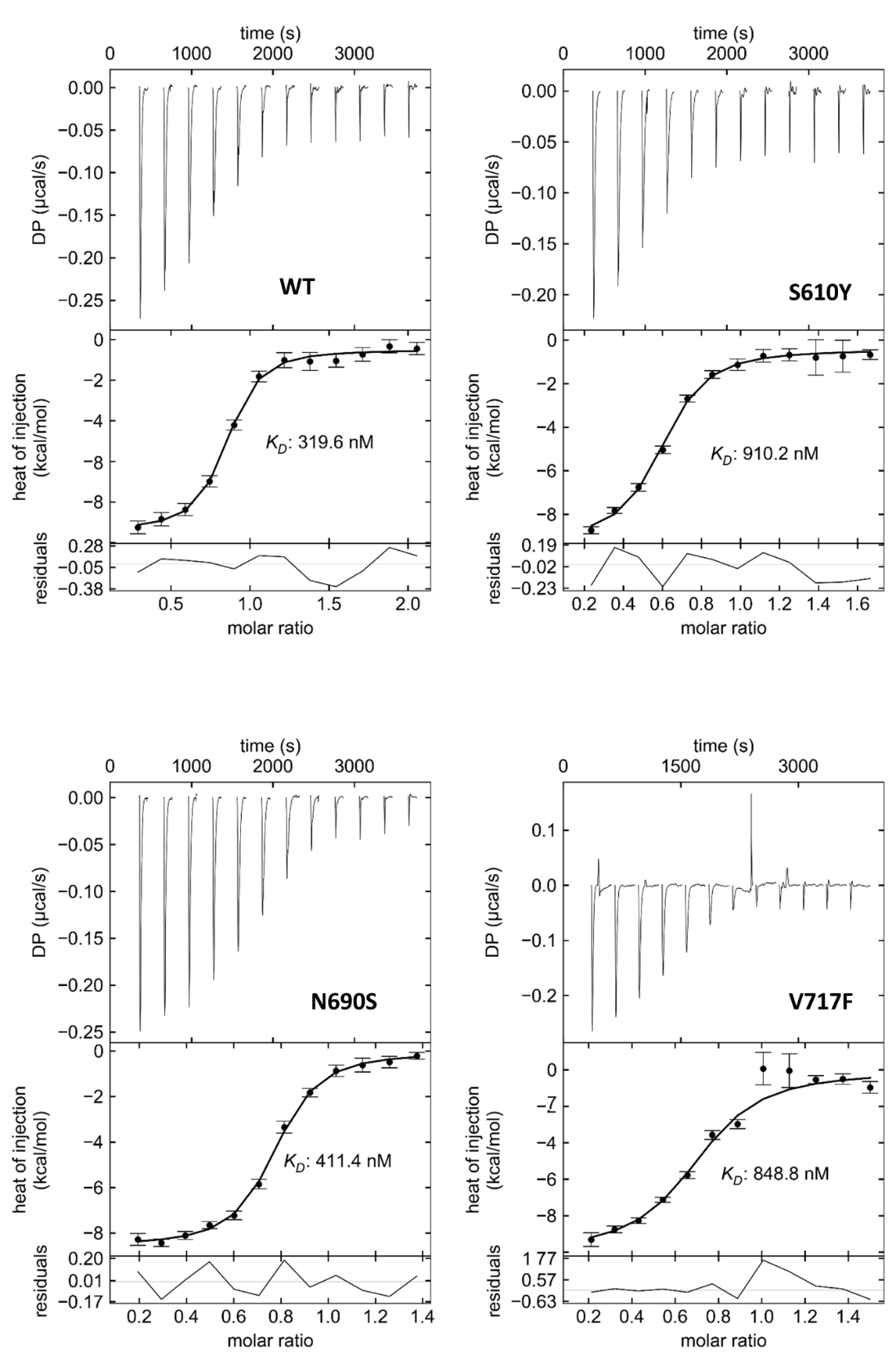


**Figure S2. Isothermal titration calorimetry.** Purified His_6_–SUMO tagged fusion proteins containing SH3 to PDZ domains of Shank2 WT and mutants were analysed with respect to binding to the synthetic peptide ADSIEIYIPEAQTRL, corresponding to the C-terminus of GKAP/SAPAP proteins. Resulting K_D_ values are shown.

**
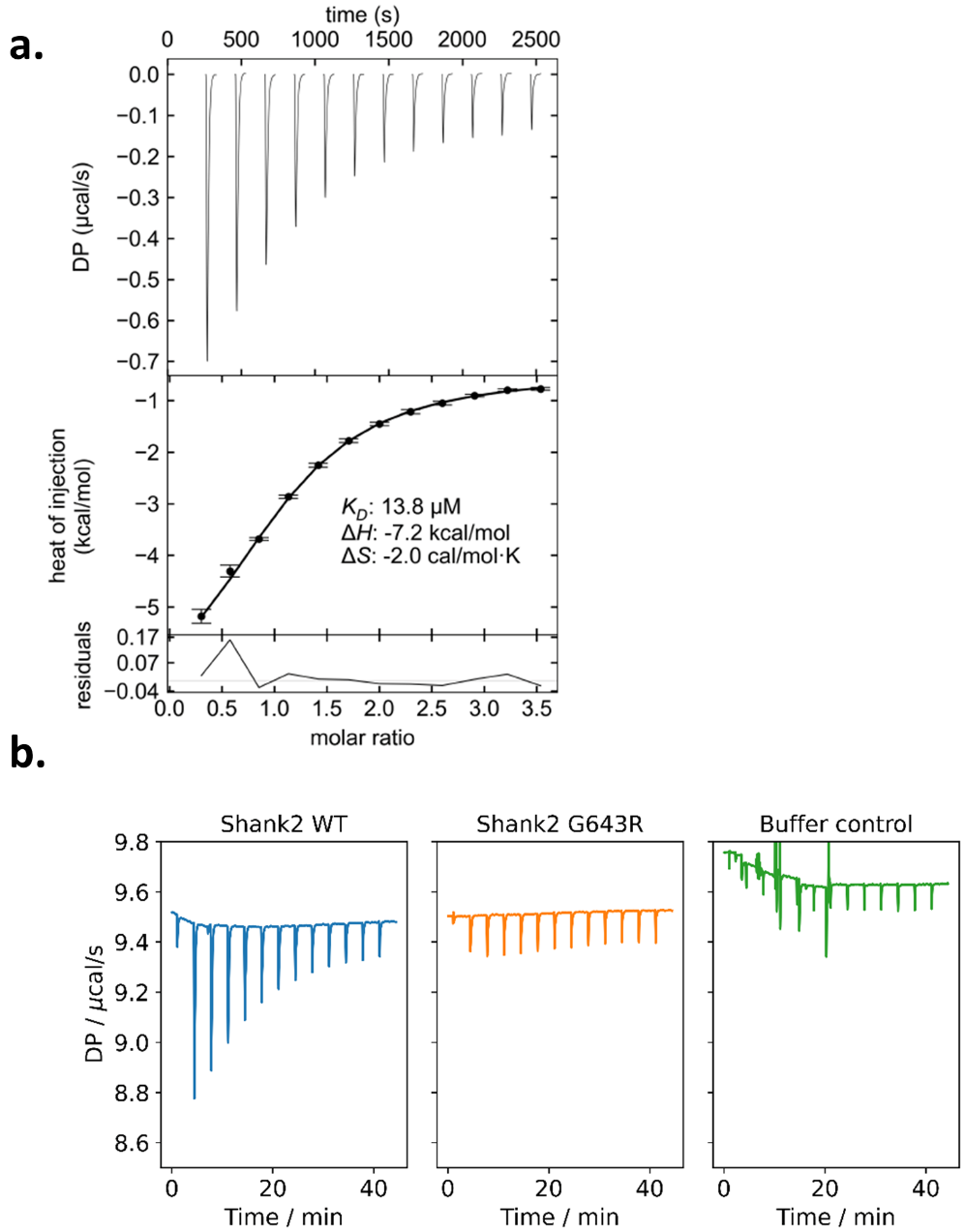
**

**Figure S3.** **a.** Isothermal titration calorimetry of purified His_6_–SUMO tagged fusion protein containing SH3 to PDZ domains of Shank2 WT vs. the synthetic peptide PGPDGDGQMQLVTSL which corresponds to the C-terminal PDZ ligand of latrophilin-1/CIRL1. A K_D_-value of 13.8 µM was calculated, 43fold lower than the K_D_ for GKAP. For the G643R mutant, no significant binding could be detected. **b.** Comparison of titration curves obtained with WT and G643R mutant protein, and buffer alone, indicating that the mutant protein behaved very similar to buffer without protein in this assay.

**
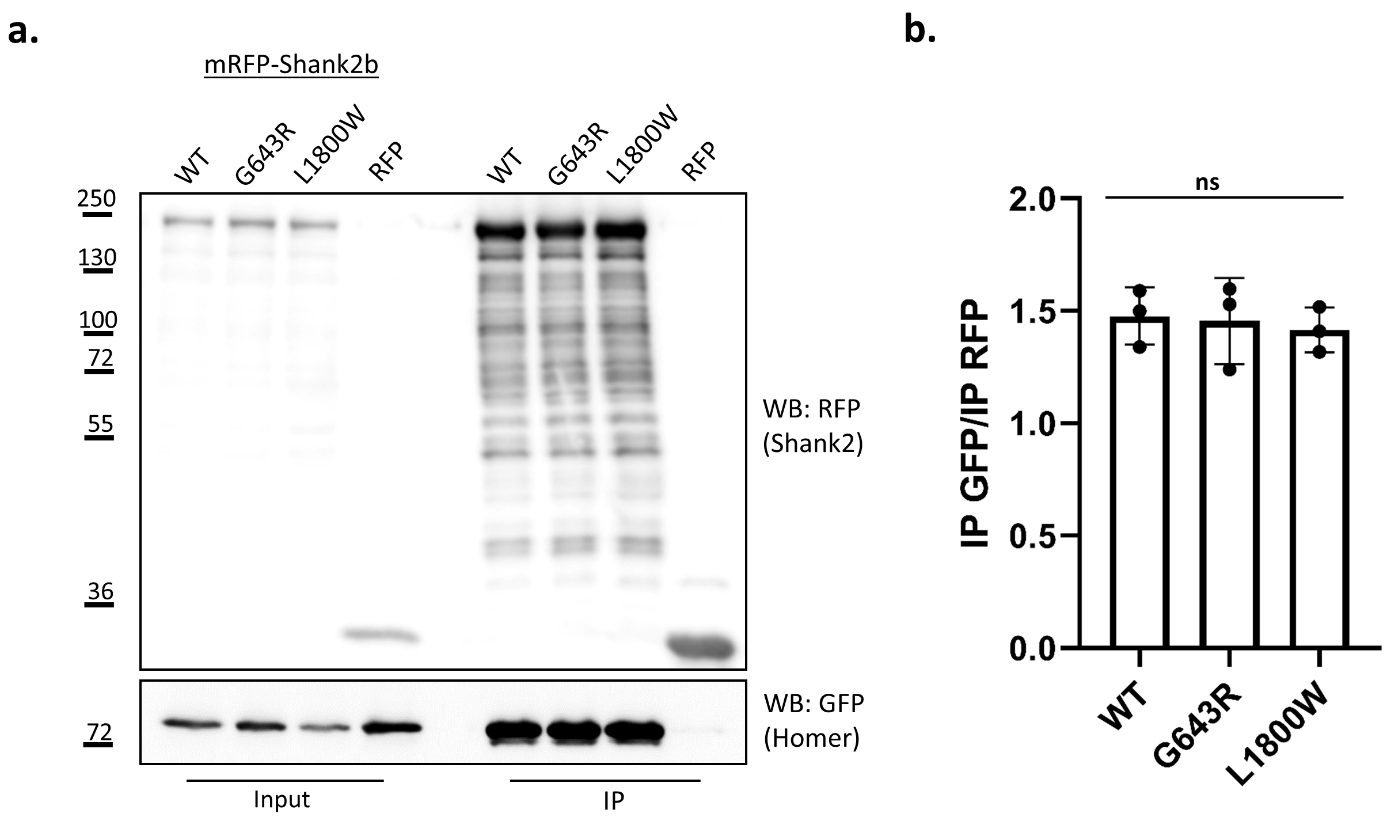
**

**Fig. S4. Mutant forms of *SHANK2* interact with Homer. a.** RFP-tagged Shank2b variants, or mRFP alone, were coexpressed in 293T cells with the GFP-tagged Homer. After cell lysis, RFP-tagged proteins were immunoprecipitated using the mRFP-trap matrix. Input and precipitate samples were analysed by Western blotting using mRFP- (upper panel) and GFP-specific antibodies (lower panel). **b**. Quantitative analysis. Signal intensities in IP samples for GFP-Homer were divided by IP signals for mRFP-Shank2b variants. ns: non-significant; data from three independent experiments; ANOVA, followed by Dunnett’s multiple comparisons test.





**Figure S5. Purification of wt and L1800W mutant Shank2 SAM domain for structural analyses.** Purified His_6_-SUMO-SAM domain fusion proteins were treated with SUMO protease overnight at 4°C in cleavage buffer (150 mM NaCl; 50 mM Tris-HCl, ph 8.0; 1 mM DTT). After cleavage, His_6_-tag containing proteins were removed from the solution by short incubation with 0.5 ml Ni-NTA matrix, followed by separation on a disposable column (Bio-Rad). Samples before and after cleavage, as well as the run through of the final column, were analysed by SDS-PAGE, followed by staining with Coomassie Brilliant Blue.

**Figure S6. Dynamic Light scattering autocorrelation functions for Shank2.** WT (red) and L1800W mutant (blue) SAM domains were measured in 150 mM NaCl; 50 mM Tris-HCl, ph 8.0; 1 mM DTT (full line) or the same buffer supplemented with 50 µM Zn^2+^ (dotted line). The wild type and mutant proteins display a similar behavior in solution in the absence of Zn^2+^. The sample is rather monodisperse (after filtering) showing one inflection point and a flat baseline. The average hydrodynamic radius is 3.7 nm. Once Zn^2+^ is added the wild type protein undergoes oligomerization/aggregation. The red dotted curve is shifted to the right confirming the existence of larger particles in suspension. The average particle size is larger than 500 nm and the profile is typical polydisperse. However, the mutant sample does not show this oligomerization/aggregation behavior after one hour of incubation with Zn^2+^. The average hydrodynamic radius is of 4.2 nm.


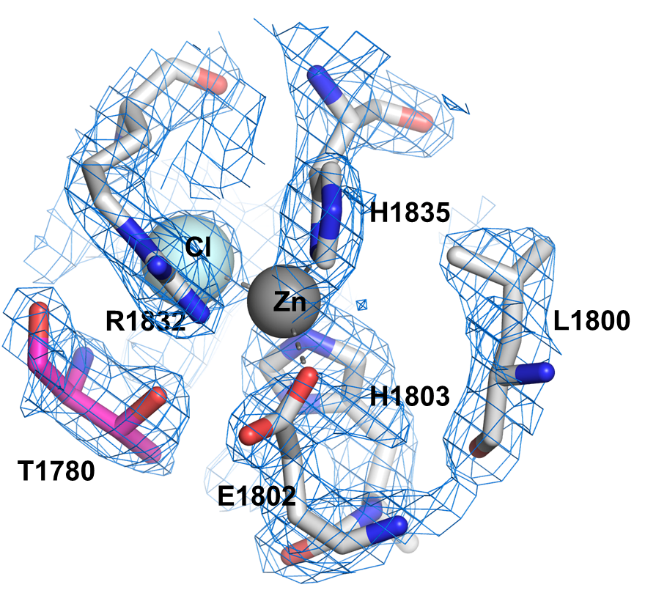


**Figure S7. Detail of the Zn^2+^ cluster in the SAM-WT structure.** The 2FoFc electron density map contoured at 1.4 sigma level is shown. Note that T1780 originates from a second SAM domain monomer, which is located in a second helical fiber of SAM domains.

**a.**


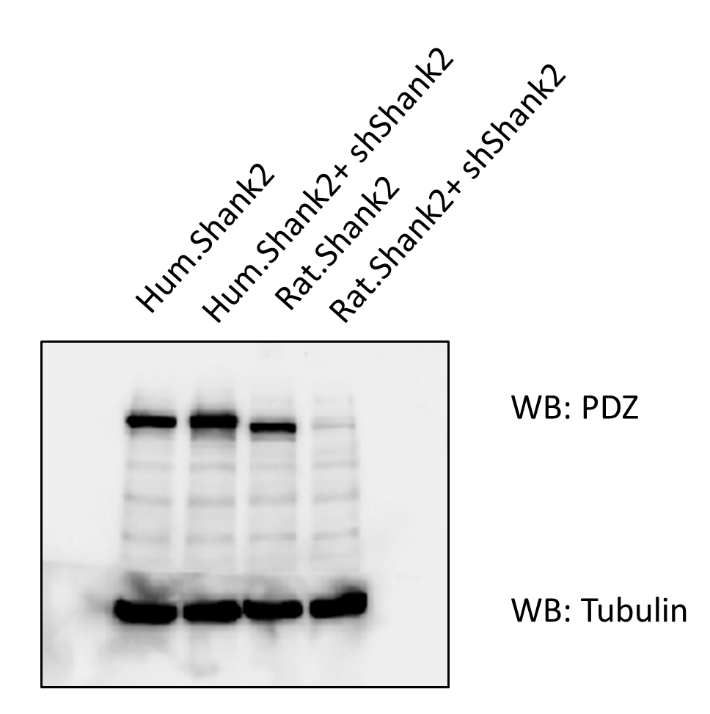


**b.**

**
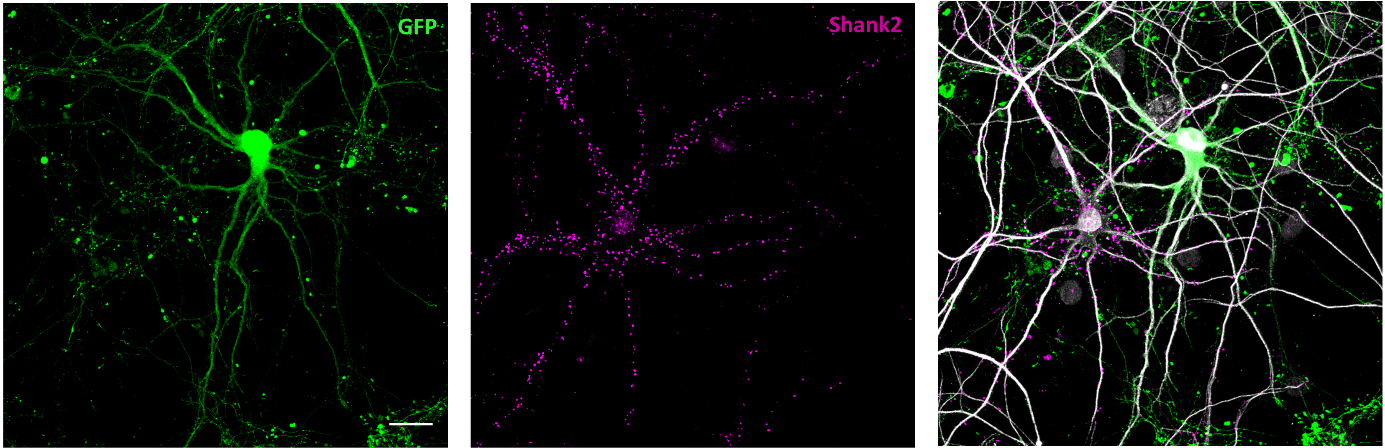
**

**Figure S8. shRNA-based knockdown of rat Shank2**. **a.** 293T cells were transfected with plasmids coding for GFP-tagged human Shank2a (left two lanes) and GFP-tagged rat Shank2b (right two lanes) with or without an shRNA vector targeting the rat Shank2 sequence, as indicated. Cells were lysed and analysed by Western blotting using anti-Shank PDZ domain antiserum (upper section of blot), or anti tubulin (lower section of blot). Note the almost complete loss of rat, but not human Shank2 upon coexpression of the shRNA. **b.** Primary cultured rat hippocampal neurons co-transfected with GFP and a shShank2 vector against the rat Shank mRNA, were stained with antibodies against Shank2 and the dendritic marker MAP2. Cells were analysed by confocal microscopy (scale bar: 20 µm).

**
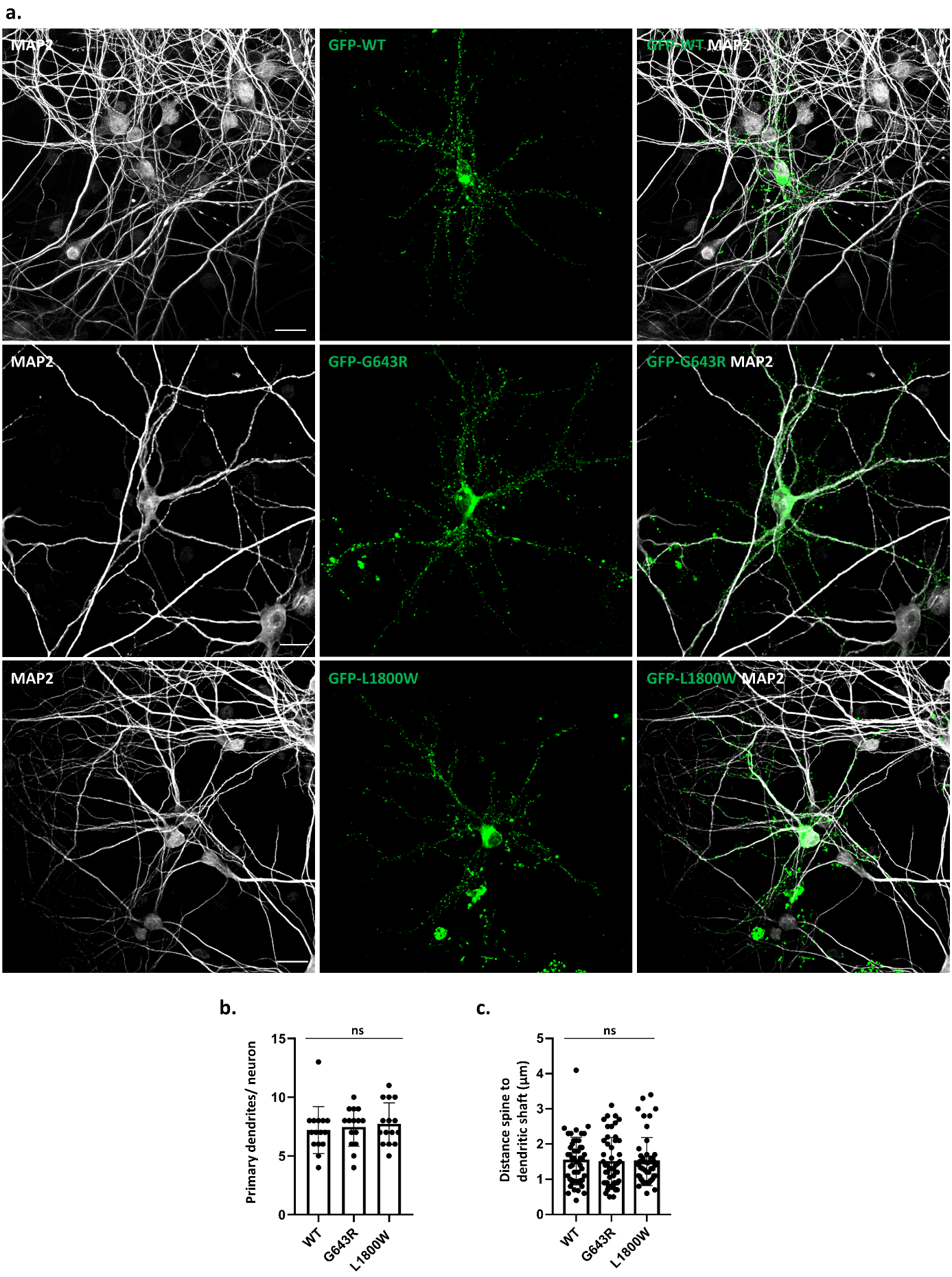
**

**Figure S9. Dendritic complexity and spine morphology are not significantly altered by mutant Shank2 variants. a.** Primary cultured rat hippocampal neurons co-transfected with GFP-tagged human Shank2 constructs and a shShank2 vector, were stained with an antibody against MAP2 (dendritic marker). Cells were analysed using confocal microscopy (scale bar: 20 µm). **b.** Quantitative evaluation of the number of primary dendrites per neuron. ns: non-significant; analysis of n = 15 neurons from three independent experiments; ANOVA, followed by Dunnett’s multiple comparison test. **c.** The distance between spine and dendritic shaft showed no difference among the three conditions. ns: non-significant; analysis of 50 spines from 10 neurons; ANOVA, followed by Dunnett’s multiple comparison test.


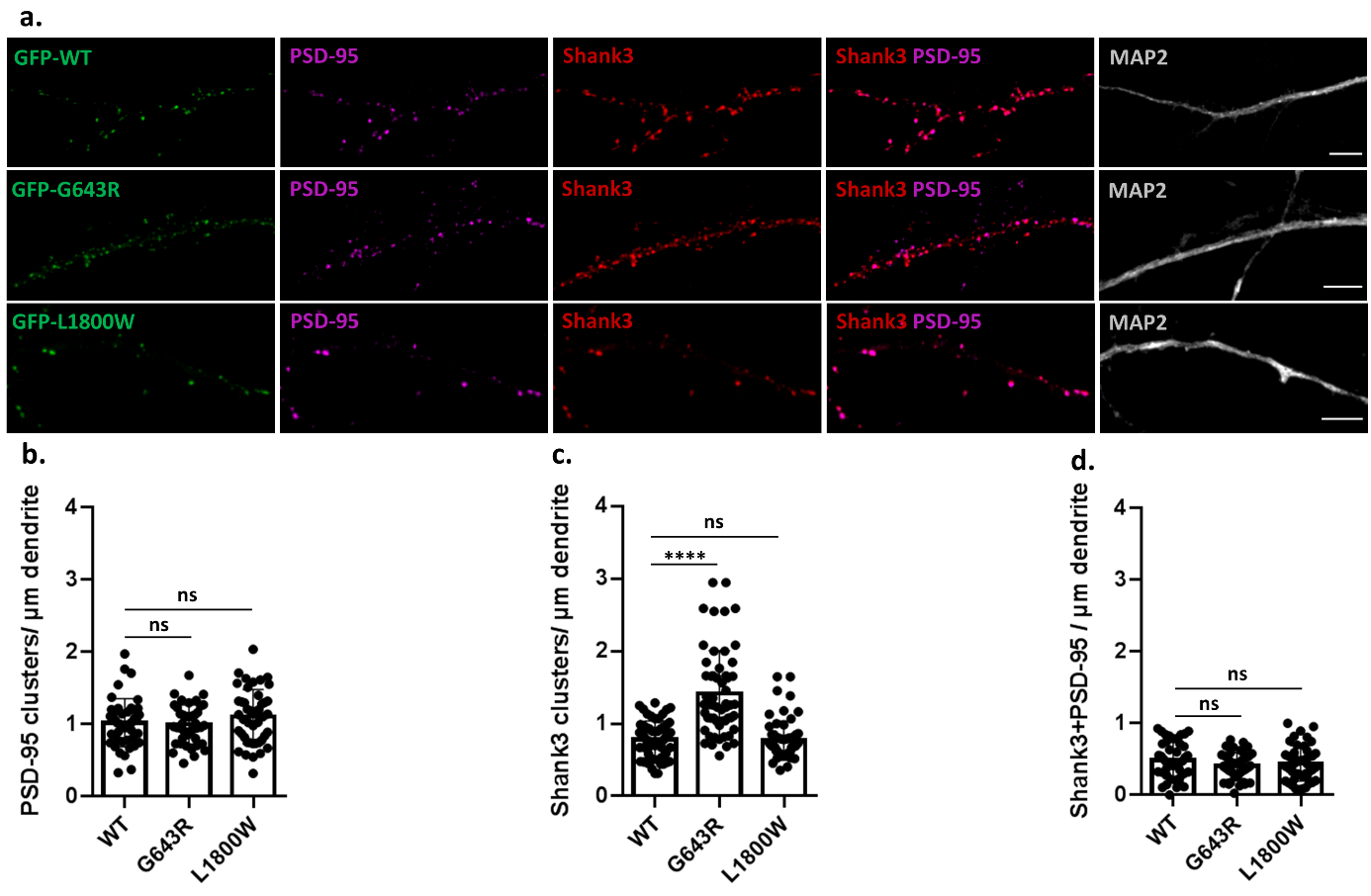


**Fig. S10 Compensatory effect of Shank3 clustering for the G643R mutant.** **a.** Primary cultured rat hippocampal neurons co-transfected with GFP-tagged human Shank2 constructs and a shShank2 vector, were stained with antibodies against PSD-95 (postsynaptic marker), MAP2 (dendritic marker) and Shank3. Cells were analysed using confocal microscopy (scale bar: 5 µm). b-d. Quantitative analysis was performed on 45 dendrites of 15 neurons from three independent experiments per each condition. **b.** The number of PSD-95 clusters per length of dendrite showed no difference among the three conditions. **c.** The number of Shank3 clusters per length of dendrite is significantly increased in neurons expressing the G643R variant of Shank2. **d.** The number of PSD-95-positive Shank3 clusters per length of dendritic branch showed no difference among the three conditions. ****: significantly different, p<0.0001; Kruskal-Wallis test, followed by Dunn’s multiple comparison test.

**
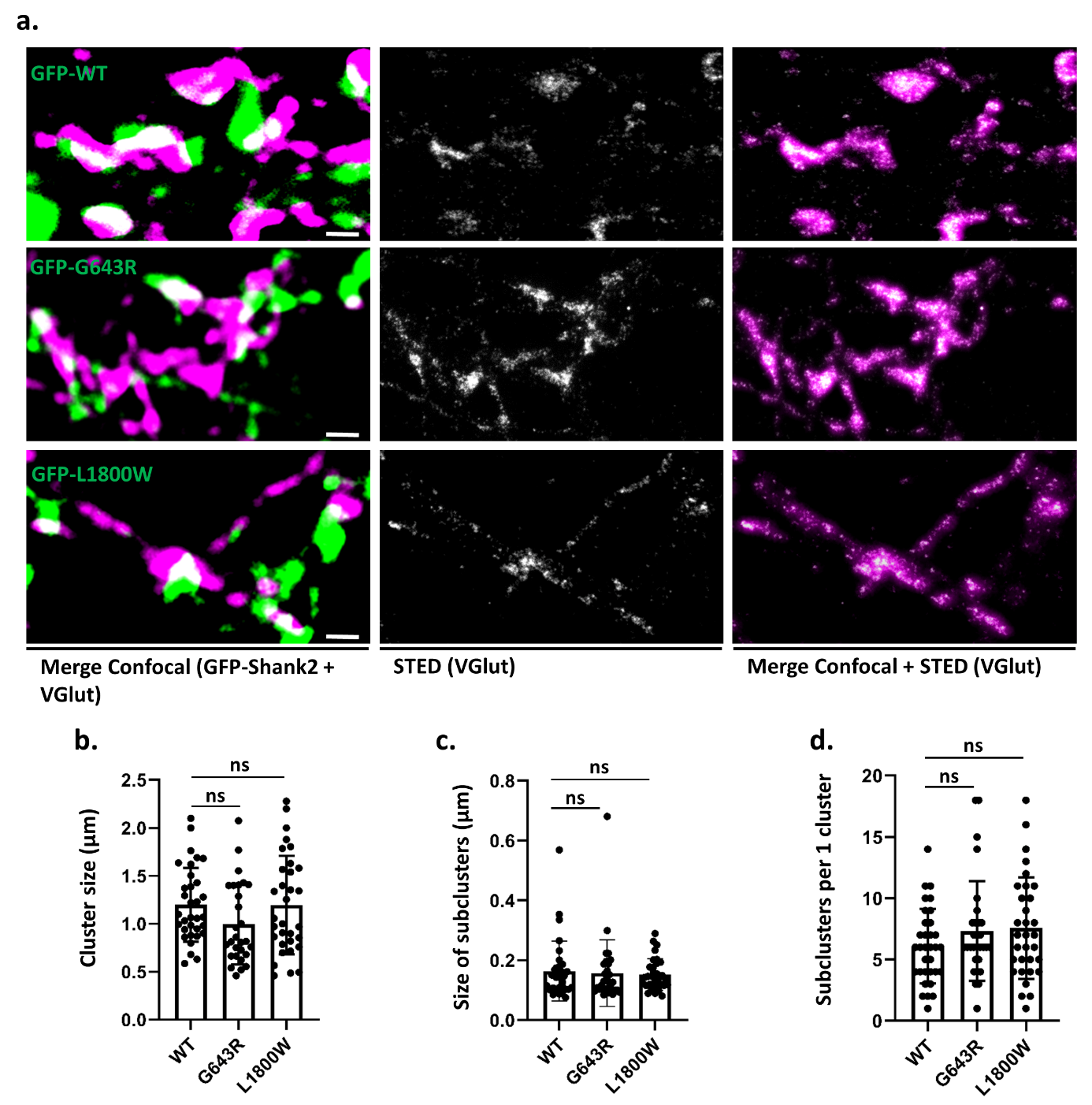
**

**Supplemental Fig. S11. Mutations in *SHANK2* do not alter the nanoscale organisation of Shank2-positive VGlut clusters. a**. Primary cultured rat hippocampal neurons co-transfected with GFP-tagged human Shank2 constructs and a shShank2 vector, were stained with an antibody against VGlut. Confocal and 2D STED imaging was performed on VGlut clusters colocalising with Shank2 (areas of colocalisation are indicated in white, left panel). Single clusters of VGlut observed in confocal mode were resolved into subclusters in superresolution mode (scale bar: 1 µm). Quantitative analysis was performed on 30 clusters from different neurons per each condition. **b.** The size of Shank2-positive VGlut clusters in the confocal mode and the size of corresponding subclusters in the superresolution mode (**c.**) did not show any difference when expressing the G643R and L1800W mutants compared to WT. **d.** Number of VGlut subclusters was not significantly altered in VGlut clusters positive for WT Shank2 compared to both mutants. ns: non-significant; ANOVA, followed by Dunnett’s multiple comparisons test.


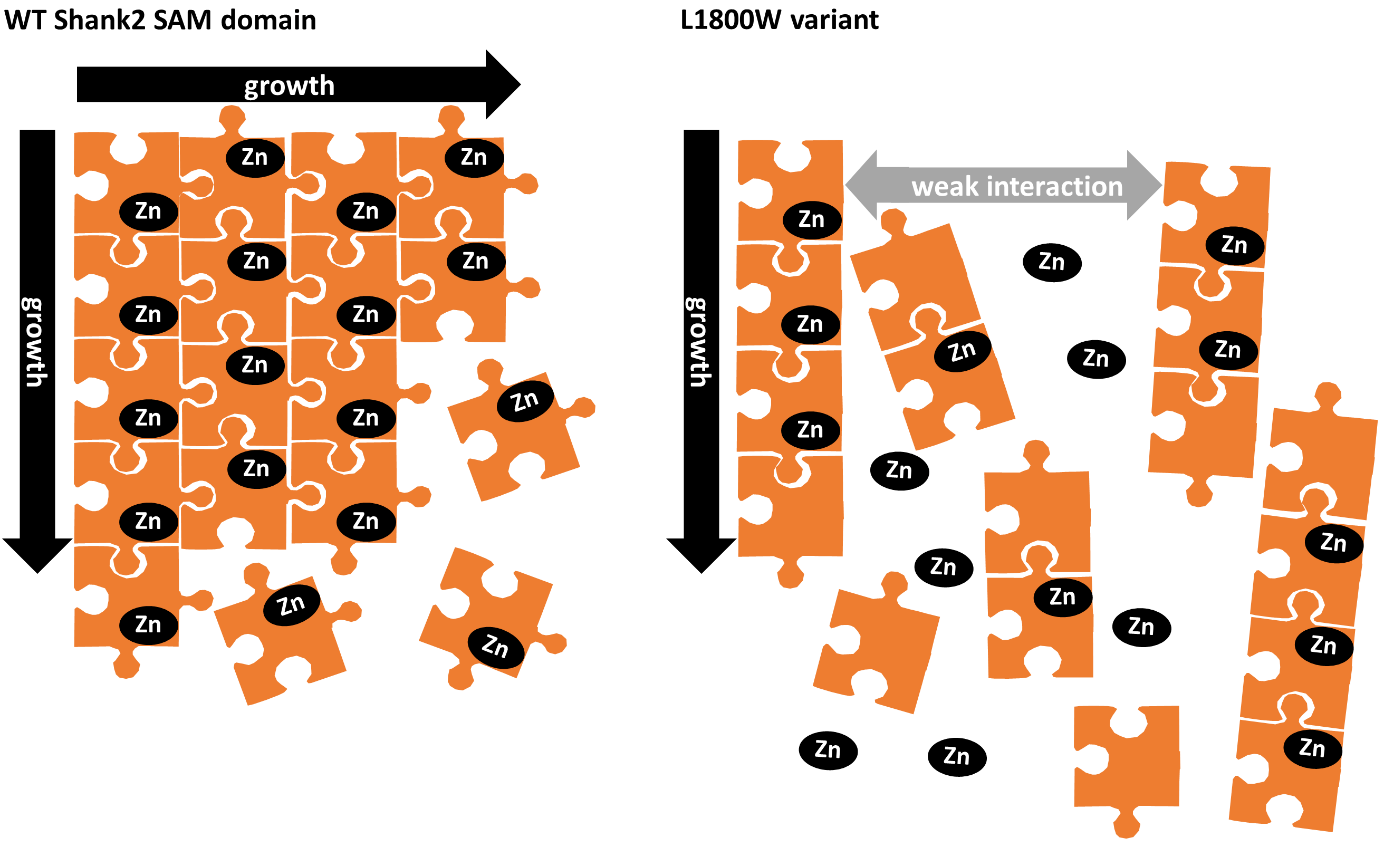


**Figure S12. Model for the effect of the L1800W mutation on SAM domain polymerization.** In the WT Shank2 SAM domain, both intrahelical and interhelical contacts between SAM domains are stabilized by the binding of Zn^2+^. Thus, in the presence of sufficient Zn^2+^, the helical sheet grows in two dimensions, as indicated. In the mutant, the affinity of the Zn^2+^ binding site is reduced. As a result, many SAM domains remain free of Zn^2+^ and can not be incorporated into growing helical fibers. Nevertheless some fibers appear to grow. In addition, side-by-side contacts between helices are weak; thus, larger clusters can only be formed once sufficiently long helices are formed, explaining the long delay in aggregate formation observed in Fig. 2g.
